# Genome-wide association study of direct oral anticoagulants and their relation to bleeding

**DOI:** 10.1007/s00228-025-03821-x

**Published:** 2025-03-21

**Authors:** Sofia Attelind, Niclas Eriksson, Mia Wadelius, Pär Hallberg

**Affiliations:** 1 Department of Medical Sciences, Clinical Pharmacogenomics, Uppsala University, Uppsala, Sweden; 2 Department of Drug Safety, Swedish Medical Products Agency, Uppsala, Sweden; 3 Uppsala Clinical Research Center, Uppsala University Hospital, Uppsala, Sweden

**Keywords:** Genome-wide association study (GWAS), Direct oral anticoagulants (DOACs), Anticoagulant-induced bleeding, Drug-related side effects and adverse reactions, *BAIAP2L2*, *CYP3A5*, *ABCG2*, *VWF*

## Abstract

**Purpose:**

Direct oral anticoagulants (DOACs) are used to prevent and treat thromboembolic events in adults. We aimed to investigate whether pharmacogenomic variation contributes to the risk of bleeding during DOAC treatment.

**Methods:**

Cases were recruited from reports of bleeding sent to the Swedish Medical Products Agency (*n* = 129, 60% men, 93% Swedish, 89% on factor Xa inhibitors) and compared with population controls (*n* = 4891) and a subset matched for exposure to DOACs (*n* = 353). We performed a genome-wide association study, with analyses of candidate single nucleotide polymorphisms (SNPs) and candidate gene set analyses.

**Results:**

Forty-four cases had major, 37 minor, and 48 clinically relevant non-major (CRNM) bleeding. When cases were compared with matched controls, *BAIAP2L2* rs142001534 was significantly associated with any bleeding and major/CRNM bleeding (*P* = 4.66 × 10^−8^ and *P* = 3.28 × 10^−8^, respectively). The candidate SNP *CYP3A5* rs776746 was significantly associated with major and major/CRNM bleeding (*P* = 0.00020 and *P* = 0.00025, respectively), and *ABCG2* rs2231142 was nominally associated with any bleeding (*P* = 0.01499). Rare coding variants in the candidate gene *VWF* were significantly associated with any bleeding (*P* = 0.00296).

**Conclusion:**

*BAIAP2L2*, *CYP3A5*, *ABCG2*, and *VWF* may be associated with bleeding in DOAC-treated patients. The risk estimates of the candidate variants in *CYP3A5* and *ABCG2* were in the same direction as in previous studies. The Von Willebrand Factor gene (*VWF*) is linked to hereditary bleeding disorders, while there is no previous evidence of bleeding associated with *BAIAP2L2.*

**Supplementary Information:**

The online version contains supplementary material available at 10.1007/s00228-025-03821-x.

## Introduction

The direct oral anticoagulants (DOACs), rivaroxaban, apixaban, and dabigatran, are used for the prevention and treatment of thromboembolic events in adults. Rivaroxaban and apixaban are inhibitors of factor Xa, while dabigatran is a direct thrombin inhibitor [1]. Despite using recommended dosing, some patients might still experience thromboembolic events or bleeding. The risk of major bleeding during treatment with a DOAC has been estimated to 2–3% per year, the risk of intracranial haemorrhage to about 0.1–0.5% per year, and the risk of major gastrointestinal bleeding to 1–1.5% [2–5].

Known factors that increase the risk of bleeding during treatment with DOACs include renal and hepatic impairment, concomitant therapy with interacting drugs, low body weight, and high age [6]. Studies indicate a potential for pharmacogenomics to help explain interindividual variation in DOAC response and the occurrence of adverse drug reactions (ADRs) [7]. A previous candidate gene study of the risk of bleeding associated with DOACs investigated eight pharmacokinetics-related single nucleotide polymorphisms (SNPs) within the genes *ABCB1*, *ABCG2*, *CYP2J2*, *CYP3A4*, and *CYP3A5* [8]. The study found no significant associations with bleeding during treatment with either rivaroxaban or apixaban. Very few genome-wide association studies (GWAS) on DOACs have been published [9]. In a GWAS investigating the effects of genetic variation on dabigatran pharmacokinetics, using data from the RELY study, rs2244613 in the *CES1* gene was associated with lower exposure to the active metabolite of dabigatran [10]. This single nucleotide polymorphism was further tested for association with clinical outcome and was found to correlate with a lower risk of bleeding [11]. In another GWAS of the pharmacokinetics of apixaban, using data from the ARISTOTLE trial, rs2231142 in *ABCG2* was associated with an increased exposure to apixaban, with a non-significant trend in risk of bleeding [12].

To our knowledge, no GWAS with the primary aim of investigating the possible association between genetic variation and the risk of bleeding has been published. We therefore conducted a GWAS, with analyses of candidate single nucleotide polymorphisms (SNPs) and candidate gene set analyses, of cases of bleeding during DOAC therapy.

## Aim

The aim was to investigate whether there is a genetic predisposition to bleeding during DOAC therapy.

## Material and methods

Patients were recruited from nation-wide spontaneous ADR reports sent from healthcare professionals to the Swedish Medical Products Agency (MPA) or by advertising, as part of the Swedegene project [13]. Reports of bleeding events associated with DOAC treatment until May 2021 were retrieved. Patients were contacted and asked to participate, and consenting patients were recruited. Participants had to be at least 18 years old at the time of recruitment, not taking other anticoagulants or strong pharmacokinetically interacting drugs, not have severe renal impairment, and be capable of providing written informed consent. The final number of cases with bleeding included in the study was 129 (Table 1).

**Table 1:** **a** Characteristics for DOAC-treated cases and controls. **b** Characteristics for DOAC-treated cases

**a**		
	Cases (*n = *129)	Controls (*n = *353)
Sex (male/female)	77/52	190/163
Mean age (years) [range]	71 [28–89]	77 [57–94]^a^
Ethnicity	93% Swedish origin 6% other European origin 1% other origin	Predominantly of Swedish genetic ancestry (>99.9%) [15]
Suspected drugs	apixaban (*n = *63) rivaroxaban (*n = *52) dabigatran (*n =* 14)	apixaban (*n = *194) rivaroxaban (*n = *91) dabigatran (*n = *68)
**b**		
All bleeding (*n = *129)	Epistaxis (*n = *44) Cerebral haemorrhage (*n = *28) Gastrointestinal haemorrhage (*n = *21) Haematuria (*n = *7) Rectal haemorrhage (*n = *6) Gynaecological haemorrhage (*n = *5) Cerebellar haemorrhage (*n = *4) Haematoma (*n = *4) Haemarthrosis (*n = *3) Other haemorrhage (*n = *7)	
Major bleeding (*n = *44)	Cerebral haemorrhage (*n = *28) Cerebellar haemorrhage (*n = *4) Haemarthrosis (*n = *3) Gastrointestinal haemorrhage (*n = *3) Haematoma (*n = *2) Rectal haemorrhage (*n = *1) Epistaxis (*n = *1) Other haemorrhage (*n = *2)	
Clinically relevant non-major bleeding (*n = *48)	Gastrointestinal haemorrhage (*n = *17) Epistaxis (*n = *8) Haematuria (*n = *7) Rectal haemorrhage (*n = *5) Genital haemorrhage (*n = *5) Haematoma (*n = *2) Other haemorrhage (*n = *4)	
Minor bleeding (*n = *37)	Epistaxis (*n = *35) Gastrointestinal haemorrhage (*n = *1) Other haemorrhage (*n = *1)	
Concomitant drugs^b^	Metoprolol (*n = *42) Paracetamol (*n = *36) Omeprazol (*n = *25) Furosemid (*n = *24) Bisoprolol (*n = *22) Losartan (*n = *20) Atorvastatin (*n = *18) Enalapril (*n = *18) Hydrochlorothiazide (*n = *15) Metformin (*n = *15) Simvastatin (*n = *15) Budesonide (*n = *14) Candesartan (*n = *14) Cyanocobalamin (*n = *12) Calcium carbonate (*n = *12) Zopiclone (*n = *12) Cholecalciferol (*n = *11) Allopurinol (*n = *10) Amlodipine (*n = *10) Levothyroxine (*n = *10) Digoxin (*n = *9) Ramipril (*n = *9) Amiloride (*n = *8) Glyceryl trinitrate (*n = *8) Prednisolone (*n = *8) Spironolactone (*n = *8) Atenolol (*n = *7) Finasteride (*n = *7) Folic acid (*n = *7) Formoterol (*n = *7) Potassium chloride (*n = *7) Latanoprost (*n = *7) Oxycodone (*n = *7) Salbutamol (*n = *7)	
Concomitant diseases^b^	Hypertension (*n = *95) Atrial fibrillation and flutter (*n = *94) Osteoarthritis (*n = *47) Cerebrovascular accident (stroke) (*n = *25) Deep vein thrombosis (*n = *24) Cholelithiasis (*n = *23) Arthroplasty (*n = *20) Cardiac failure (*n = *20) Hyperlipidaemia (*n = *21) Pulmonary embolism (*n = *21) Diabetes mellitus (*n = *19) Asthma (*n = *18) Glaucoma (*n = *16) Nephrolithiasis (*n = *15) Prostate cancer (*n = *13) Gastric ulcer (*n = *12) Obesity (*n = *11) Psoriasis (*n = *11) Chronic obstructive pulmonary disease (*n = *9) Hypothyroidism (*n = *9) Rheumatoid arthritis (*n = *7) Eczema (*n = *7)	

^a^Age at start of treatment with DOAC (Direct Oral Anticoagulants)

^b^Showing concomitant drugs and diseases that were present in at least 5% of the cohort

Clinical data (demographics, smoking and alcohol use, medical history, history of drug treatment, and ancestry) were collected through telephone interviews using a standardised questionnaire and by obtaining and reviewing medical records. Blood samples were drawn at the patient’s nearest healthcare facility and were sent to our laboratory where they were stored at − 70 °C.

All cases of bleeding were categorised as major, minor, or clinically relevant non-major bleeding (CRNM) depending on the type and location of the bleeding [3, 14]. Each case was adjudicated by two specialists in clinical pharmacology and drug safety.

A total of 4891 unrelated individuals from the Swedish TwinGene biobank, predominantly of Swedish genetic ancestry and born between 1911 and 1958, were used as population controls [15]. A subgroup matched for DOAC exposure was also used (*n* = 353). This information was obtained through linkage with the Swedish Prescribed Drug Register.

Analyses were performed using both all population controls and controls matched for DOAC exposure. We analysed both all cases of bleeding and stratified by major or major/CRNM bleeding. The analyses were also stratified by type of DOAC (factor Xa inhibitors or direct thrombin inhibitor). Subgroup analyses were performed for apixaban and rivaroxaban when results were statistically significant for factor Xa inhibitors.

### Genome-wide array data and quality control

DNA was extracted from peripheral venous blood. Genotyping for cases was done with the Illumina Infinium OmniExpressExome-8 v1.3 or Infinium OmniExpressExome-8 v1.6 and for controls with the Illumina Human OmniExpress 700 K array. Genotype calls were generated using the Genome Studio software from Illumina.

Genome-wide genotyping quality control (QC) and data management were performed using PLINK v. 1.9. QC included check of sex and SNPs, and individuals were included based on per SNP call rate and individual call rate > 98%, minor allele frequency (MAF) > 0.005 and Hardy–Weinberg equilibrium *P*-value > 5 × 10^−8^. In addition, the data was checked versus the haplotype reference consortium data (HRC v1.1) for strand issues. After the QC, the data was merged, and imputation of genotypes was performed using the TOPMED reference panel and the Michigan imputation server. Post-imputation, the data was filtered on imputation quality (mimimac imputation quality R2 > 0.7) and converted to hard calls using PLINK. The total number of SNPs after imputation and filtering was 24,049,323. Genetic principal components were estimated using PLINK v1.9 on the non-imputed data. In addition, principal components were estimated on the non-imputed data when merged with Hapmap (release 23, 270 individuals).

All results are reported in accordance with the Genome Reference Consortium human assembly GRCh38.

### Candidate variants and genes

Candidate SNPs were selected based on previous associations with bleeding related to DOACs (Table 2). Candidate genes were selected based on the pharmacokinetics of DOACs and based on the ISTH list of genes associated with bleeding [16] (Table 3). The candidate SNPs and genes were prespecified in the statistical analysis plan prior to statistical analyses.

**Table 2:** List of candidate single nucleotide polymorphisms

**Gene and variant [ref]**	**Consequence**	**SNP**	**Chr**	**Position, base pair**^a^	**Minor allele**^b^	**Major allele**^b^
ABCB1 c.3435 [28, 29]	Missense variant [30, 31]	rs1045642	7	87509329	G	A
ABCB1 c.1236 [28, 29]	Synonymous variant [30–32]	rs1128503	7	87550285	A	G
ABCG2 c.421 [28, 33]	Missense variant, stop gained [34, 35]	rs2231142	4	88131171	T	G
ABCG2 c.34 [36]	Missense variant [30, 31]	rs2231137	4	88139962	T	C
CYP3A5 *3 [28, 33]	Intron splice variant [34, 35]	rs776746	7	99672916	T	C
CYP3A4 *22 [37]	Intron splice variant [34, 35]	rs35599367	7	99768693	A	G
CYP3A4 *1B [37]	5-prime flanking promoter variant [34, 35]	rs2740574	7	99784473	C	T
CES1 [10]	Intron variant associated with reduced enzyme activity [10, 38]	rs2244613	16	55810697	G	T
CES1 [10]	Intron variant associated with reduced enzyme activity [10, 39]	rs8192935	16	55827882	A	G

*SNP*, single nucleotide polymorphism; *Chr*, chromosome

^a^All results are reported in accordance with the Genome Reference Consortium human assembly GRCh38

^b^Reported on the forward strand

**Table 3:** List of candidate genes for DOAC bleeding. Selected due to the pharmacokinetics of DOACs and from the ISTH list of genes associated with bleeding [16]

Gene [ref]	Chromosome
SULT1A1 [40]	16
ABCB1 [28, 29]	7
ABCG2 [33]	4
CYP3A4 [41]	7
CYP3A5 [28, 33]	7
CES1 [10]	16
CES2 [42]	16
F12 [16]	5
FGA [16]	4
FGB [16]	4
FGG [16]	4
GGCX [16]	2
SERPINE1 [16]	7
SERPINF2 [16]	17
THBD [16]	20
VWF [16]	12

### Statistical analyses

All analyses were performed using SAIGE (Scalable and Accurate Implementation of GEneralized mixed model [17]) with Firth’s Bias-Reduced estimates for variants with *P*-values below 0.05. In short, SAIGE handles case–control imbalance and bias in results due to low frequency variants. All genome‐wide analyses were adjusted for sex and the first four principal components. SNP effects were modelled as additive. Results are presented as Manhattan plots.

Power calculations for GWAS using an additive genetic model showed that 100 cases and 5000 controls gave 80% power to detect an odds ratio (OR) of 3–4. This was based on a MAF of 10–20%, and a significance level of *P* < 5 × 10^−8^ (Fig. S1 in the Online Resource). The power calculations were performed using the function GPC from the R-package GeneticDesign.

The significance level for the analyses was set according to a pre-defined order of hypothesis testing where the order was candidate SNPs, candidate gene set analyses, and GWAS. The significance levels for the candidate SNP and gene set analyses were set at the Bonferroni corrected threshold according to the number of tests in each stage. This was *P* < 0.05/9 = 0.00556 for the candidate SNPs and *P* < 0.05/16 = 0.003125 for the candidate gene set analyses. In the GWAS analyses, the conventional genome‐wide significance threshold *P* < 5 × 10^−8^ was used.

For the candidate gene set analyses, we included non-synonymous missense variants. These variants were tagged according to dbsnp 153 (hg38) by using the tag maxFuncImpact id = 1583 for missense variant. Five MAF cutoffs were used in the gene set analyses, 0.001, 0.01, 0.1, 0.2, and 0.4. Statistics and *P*-values for the sequence kernel association test (SKAT) and Burden tests are presented. The primary test in the gene set analyses was the SKAT-O test which is a combination of the SKAT and Burden tests.

## Results

A total of 138 cases were recruited, of which nine were excluded for the following reasons: fall trauma before ADR onset (*n* = 2), DOAC not suspected to be the causative drug (*n* = 1), multiple suspected drugs (*n* = 1), surgery preceding the ADR (*n* = 1), insufficient medical information available (*n* = 3), and did not pass genotyping QC (*n* = 1). A total of 129 cases thus remained, and the characteristics of the patients are shown in Tables 1a and b. Most had factor Xa inhibitors as the suspected drug (apixaban (*n* = 63) or rivaroxaban (*n* = 52)), while 14 cases received the direct thrombin inhibitor dabigatran. Most, 93%, were of Swedish origin, 6% of other European origin, and the remaining 1% of other origins. A plot of the first two principal components when the data was merged with Hapmap is presented in Supplementary Fig. S2. There were no major deviations from the European cluster for the ADR cases. There were more men than women (60% vs 40%). The type of bleeding included gastrointestinal, cerebral, intraocular, intraarticular, intramuscular, urologic, gynaecologic, and pulmonary bleeding, and epistaxis. The cases of bleeding were categorised as major (*n* = 44), minor (*n* = 37), or clinically relevant non-major bleeding (CRNM) (*n* = 48).

### Genome-wide association analyses

**Fig. 1:**
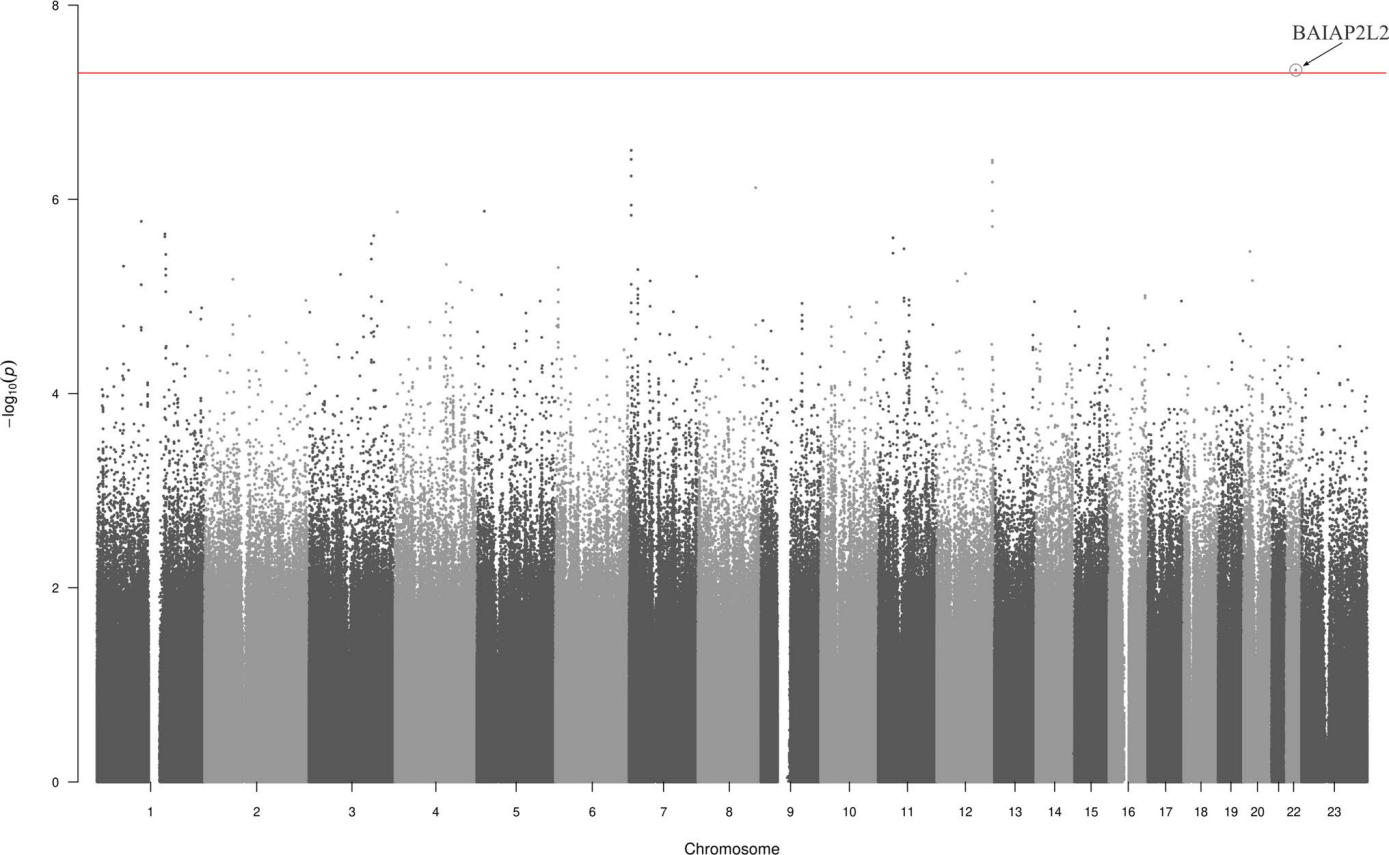
Manhattan plot of the genome-wide association study including all cases of bleeding (*n* = 129) vs population controls matched for DOAC exposure (*n* = 353), adjusted by sex and genetic principal components 1–4. The horisontal line denotes the genome-wide significance level *P* < 5 × 10^−8^. One SNP passed the significance threshold, rs142001534 located in BAR/IMD domain containing adaptor protein 2 like 2 (*BAIAP2L2*) on chromosome 22, (OR 12.1 [95% CI 4.3–34.2], *P* = 4.66 × 10^−8^)

No genetic marker passed the significance threshold when the full data set including all cases and controls was used (Fig. S3, Table S1 in the Online Resource). However, when cases were compared with controls matched for treatment with a DOAC, one SNP in the gene BAR/IMD domain containing adaptor protein 2 like 2 (*BAIAP2L2*) [18] on chromosome 22 passed the significance threshold, rs142001534 (Fig. 1 and Table S2 in the Online Resource), OR 12.1 [95% CI 4.3–34.2], *P* = 4.66 × 10^−8^. The same was seen when analysing only cases with major or CRNM bleeding (*P* = 3.28 × 10^−8^, Table S3 and Fig. S4 in the Online Resource), but in none of the other subgroup analyses.

### Candidate variant analyses of pre-specified variants

**Fig. 2:**
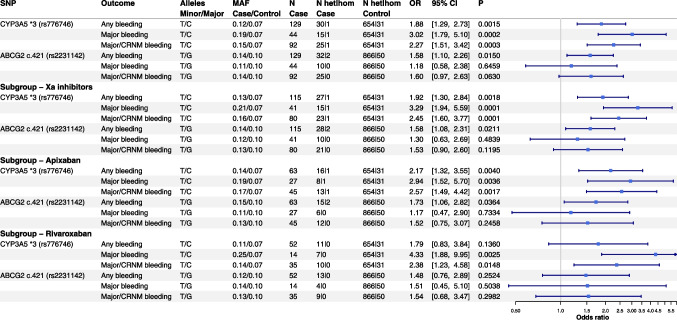
Forest plot of the candidate variants *CYP3A5* rs776746 and *ABCG2* rs2231142 including all cases of bleeding (*n* = 129), major bleeding (*n* = 44), and major plus clinically relevant non-major bleeding (CRNM) (*n* = 92) vs all population controls (*n* = 4891). Also showing the same for the largest subgroup factor Xa inhibitors and for apixaban and rivaroxaban separately. *SNP*, single nucleotide polymorphism; *MAF*, minor allele frequency; *N*, het/hom, number of heterozygotes and homozygotes for the minor allele; *OR*, odds ratio with lower and upper 95% confidence interval (CI)

The *CYP3A5* gene is located on the reverse strand of the chromosome 7. The candidate variant rs776746A that encodes a functional cytochrome P450 3A5 enzyme was significantly associated with bleeding in all candidate variant analyses (Table 4), except for the subgroup treated with dabigatran. The strongest association was seen when cases with major bleeding during factor Xa inhibitor treatment were compared with matched controls (OR 4.6 [95% confidence interval (CI) 2.3–9.2], *P* = 2.46 × 10^−5^) (Table S4 in the Online Resource). The *ABCG2* variant rs2231142, previously associated with increased exposure to apixaban [12], was nominally associated with bleeding (OR 1.6, *P* = 0.0150, all cases vs all controls, Fig. 2).

**Table 4:** Candidate variant results for cases of bleeding due to DOACs (apixaban, rivaroxaban, dabigatran)

CHR	Position^a^	SNP	Alleles minor/major	MAF cases	MAF controls	N cases/ controls	OR [95% CI]	*P*	Gene
7	99672916	rs776746^b^	T/C	0.124	0.073	129/4891	1.877 [1.291, 2.729]	1. 503 × 10^−3^*	CYP3A5
4	88131171	rs2231142	T/G	0.14	0.099	129/4891	1.578 [1.103, 2.259]	0.01498823	ABCG2
7	99784473	rs2740574	C/T	0.062	0.039	129/4891	1.861 [0.986, 3.513]	0.05540575	CYP3A4
7	99768693	rs35599367	A/G	0.05	0.032	129/4891	1.732 [0.865, 3.469]	0.1211466	CYP3A4
16	55827882	rs8192935	A/G	0.291	0.32	129/4891	0.877 [0.673, 1.145]	0.3350927	CES1
4	88139962	rs2231137	T/C	0.031	0.043	129/4891	0.756 [0.401, 1.422]	0.3854012	ABCG2
7	87509329	rs1045642	G/A	0.45	0.421	129/4891	1.102 [0.86, 1.413]	0.441856	ABCB1
16	55810697	rs2244613	G/T	0.159	0.176	129/4891	0.879 [0.631, 1.224]	0.4438239	CES1
7	87550285	rs1128503	A/G	0.422	0.431	129/4891	0.949 [0.74, 1.218]	0.6813987	ABCB1

*CHR*, chromosome; *SNP*, single nucleotide polymorphism; *MAF*, minor allele frequency; *N*, is the number of non-missing values; *OR*, odds ratio with lower and upper 95% confidence interval (CI). *The *P*-value for significance was set to 0.00556, according to Bonferroni adjustment for the number of tested SNPs (*n* = 9)

^a^All results are reported in accordance with the Genome Reference Consortium human assembly GRCh38

^b^Please note that CYP3A5 is read on the reverse strand, i.e. rs776746 T/C is A/G in the gene’s sense strand. All results are reported on the forward strand

Figure 2 shows the results for *CYP3A5* rs776746 and *ABCG2* rs2231142 in the apixaban and rivaroxaban subgroups. Results for *CYP3A5* rs776746 were overall similar for apixaban and rivaroxaban, while there was a trend towards a stronger signal for *ABCG2* rs2231142 in the apixaban subgroup.

### Candidate gene set analyses of pre-specified genes

Coding variants in the *Von Willebrand factor* (*VWF*) gene on chromosome 12 were significantly associated with bleedings in the candidate gene set analyses when all cases were compared with all population controls (*P* = 0.00296) (Table 5a and Table S5 in the Online Resource). This was also true when the analysis was restricted to factor Xa inhibitors compared with all controls (all bleedings and major or CRNM bleeding) (*P* = 0.00171 and *P* = 0.00208, Table S6 in the Online Resource). Subgroup analyses for apixaban and rivaroxaban revealed that rare variants in *VWF* were equally distributed in both groups, and none of the analyses were significant (data not shown). The top single SNP providing information to the *VWF* gene set analysis was rs192374602 with OR 46.4 [95% CI 3.8–561], *P* = 2.47 × 10^−2^ (Table 5b).

**Table 5:** **a** Candidate gene set results for all cases of bleeding (*n = *129) vs all controls (*n = *4891). **b** Variants contributing to the gene set analysis of VWF with MAF cutoff of 0.001. Showing only variants present in both cases and controls

**a**												
Region	CHR	max MAF	*P* value SKAT-O	*P* value Burden	*P* value SKAT	BETA Burden	SE Burden	OR [95% CI]	MAC case	MAC control	Number rare	Number ultra rare
VWF	12	0.001	0.00296*	0.00296	0.00296	0.10360	0.0349	1.11 [1.04, 1.19]	5	41	0	18
VWF	12	0.01	0.01726	0.20006	0.00962	0.02289	0.0179	1.02 [0.99, 1.06]	9	206	6	18
VWF	12	0.1	0.17713	0.13455	0.12192	0.01589	0.0106	1.02 [1.00, 1.04]	101	3816	15	18
VWF	12	0.2	0.17484	0.13123	0.12328	0.01577	0.0105	1.02 [1.00, 1.04]	167	6199	17	18
VWF	12	0.4	0.17484	0.13123	0.12328	0.01577	0.0105	1.02 [1.00, 1.04]	361	13470	19	18
**b**												
CHR	Position^a^	SNP	Alleles minor/major	MAF cases	MAF controls	*N* cases/ controls	*N* cases het/hom	*N* controls het/hom	BETA Burden	SE Burden	OR [95% CI]	*P*
12	5994421	rs192374602	C/G	0.0039	2 × 10^-4^	129/4891	1/0	2/0	3.837	1.272	46.4 [3.8, 561]	0.02468647
12	5994547	rs779004919	T/C	0.0039	1 × 10^-4^	129/4891	1/0	1/0	3.701	1.426	40.5 [2.5, 662]	0.02634938
12	6031464	rs1447571715	A/T	0.0039	1 × 10^-4^	129/4891	1/0	1/0	3.590	1.419	36.2 [2.2, 584]	0.02863695
12	5994084	rs754719008	C/T	0.0039	1 × 10^-4^	129/4891	1/0	1/0	3.503	1.418	33.2 [2.1, 535]	0.03096334
12	5991849	rs200300292	G/C	0.0039	1 × 10^-4^	129/4891	1/0	1/0	3.481	1.421	32.5 [2.0, 526]	0.03197179

*CHR*, chromosome; *SNP,* single nucleotide polymorphism; *MAF*, minor allele frequency; *N* is the number of non-missing values; *BET*A, the beta coefficient is the degree of change in the outcome variable for every unit of change in the predictor variable; *SE*, standard error; *OR*, odds ratio with lower and upper 95% confidence interval (CI); *max MAF*, this is the cutoff for the maximum MAF of the variants used in the gene set analysis; *SKAT*, sequence kernel association test; *MAC*, minor allele count. *Number rare*, the number of variants with MAC above 10 in total; *Number ultra rare*, the number of variants with MAC below or equal to 10. These are combined in the analysis

^**a**^All reported in accordance with the Genome Reference Consortium human assembly GRCh38

*The *P*-value for significance was set to 0.003125, according to Bonferroni adjustment for the number of tested genes (*n = *16)

## Discussion

The results of this study suggest that variants affecting function of *BAIAP2L2*, *CYP3A5*, and *VWF* may be associated with bleeding in patients treated with DOACs, in particular apixaban and rivaroxaban.

The protein encoded by *BAIAP2L2* binds phosphoinositides and promotes the formation of planar or curved membrane structures [18]. It is mostly expressed in the gastrointestinal tract and the kidneys [19] and has not previously been shown to be associated with bleeding or altered coagulation. It is predicted to interact with several proteins involved with clathrin-mediated endocytosis [20]. Clathrin is a protein involved in the formation of coated vesicles, important for intracellular trafficking and endocytosis in cells, including platelets [21, 22]. *BAIAP2L2* rs142001534 is a missense variant that is not in linkage disequilibrium with any nearby SNP. In addition, the allele frequency was lower in the matched control group compared to all controls. We can thus not exclude a chance finding.

The enzymes CYP3A4 and CYP3A5 are major metabolisers of factor Xa inhibitors, but not of dabigatran [1]. Of the two enzymes, CYP3A5 is more strongly influenced by genetic factors. Most Europeans are non-expressors of CYP3A5 due to a common change from A to G at position 6981 from the start of transcription (rs776746G) [23]. This splice variant is less common in Africans, who more frequently carry the functional rs776746A variant [24]. In our study, the non-functional rs776746G allele had a frequency of 92.7% among population controls, aligning with European data [23]. However, we observed a higher proportion of the functional rs776746A allele in DOAC-associated bleeding cases. Campos-Staffico et al. also noted a tentative link between *CYP3A5* rs776746 AA carriers and an increased risk of bleeding from DOACs (*P* < 0.001) [8], though the small number of rs776746 AA carriers (*n* = 8) suggests this might be due to chance. Since factor Xa inhibitors are metabolised by CYP3A5 with no known active metabolites, carriers of the functional ‘A’ allele that encodes an active enzyme would be expected to have lower active drug plasma concentrations and thus a reduced bleeding risk compared to those with the ‘G’ allele. However, in a study by Wu et al., *CYP3A5* rs776746 had no clinical effect on rivaroxaban concentrations [25], and in a study by Skripka et al., *CYP3A5* rs776746 had no association with apixaban exposure [26]. Thus, our and Campos-Staffico’s findings [8] therefore warrant further investigation.

Von Willebrand factor (VWF) is a critical glycoprotein that plays a key role in blood coagulation by facilitating platelet adhesion and stabilising coagulation factor VIII. Deficiencies in VWF, whether quantitative or qualitative, lead to von Willebrand’s disease (VWD), the most common inherited bleeding disorder [27]. In our study, rare coding variants of *VWF* were significantly associated with bleeding due to all DOACs and with Xa inhibitors. None of the cases had a known diagnosis of VWD, and none of the detected variants have, to our knowledge, previously been associated with VWD.

In our previous candidate gene analyses of the pharmacokinetics of apixaban, including 1325 participants, the drug transporter gene *ABCG2* (c.421G > T, rs2231142) was significantly associated with increased exposure to apixaban [12]. On average, heterozygotes displayed a 5% increase in AUC at steady state and homozygotes a 17% increase in AUC, compared with homozygotes for the wild-type allele. Individuals carrying the rs2231142 variant had a non-significantly increased hazard ratio (HR) for major bleeding and haemorrhagic stroke (HR 1.2 and 1.9, respectively). In the current study, the OR for bleeding was 1.6, in carriers of the rs2231142 variant, but this was not statistically significant after correction for multiple testing.

There are some limitations to consider in this study. First, we were able to include 129 patients, and the power to detect associated variants was thus limited. Second, almost all participants were of white European ancestry, and thus, the results cannot be generalised to other ancestries.

## Conclusions

*BAIAP2L2*, *CYP3A5*, *ABCG2*, and *VWF* may be associated with bleeding in DOAC-treated patients. Certain variants of *VWF* are known to cause a hereditary bleeding disorder. The association with *CYP3A5* rs776746A is less straightforward, and replication studies are needed to evaluate this finding. This could be done in patients of African ancestry, in whom the ‘A’ allele is much more frequent. The variant *ABCG2* rs2231142 was linked to increased exposure to apixaban [12] in a previous study and tended to be associated with DOAC bleeding. These findings need replication in further studies.

## Competing Interests

SA is employed by the Swedish Medical Products Agency, SE-751 03 Uppsala, Sweden. The views expressed in this paper are the personal views of the authors and not necessarily the views of the Government agency. NE reports institutional research grant from Bristol-Myers Squibb/Pfizer. MW and PH declare that they have no conflict of interest.

## Supplementary Information

Below is the link to the electronic supplementary material.

Supplementary file1 (PDF 1704 KB)

## Data Availability

The datasets generated and analysed during the current study are not publicly available due to the European General Data Protection Regulation (GDPR), which requires us to protect the identity of participants, but datasets are partly available from the corresponding author on reasonable request.
