# Supplementary material for: Genome-wide association study of direct oral anticoagulants and their relation to bleeding
Source: Eur J Clin Pharmacol. 2025 Mar 21;81(5):771–83. doi: 10.1007/s00228-025-03821-x (PMC12003525; doi:10.1007/s00228-025-03821-x)
Supplement: Supplementary file 1 — Supplementary file1 (PDF 1704 KB) [file 228_2025_3821_MOESM1_ESM.pdf]

Supplementary Material

Genome-Wide Association Study of Direct Oral Anticoagulants and Their Relation to Bleeding

European Journal of Clinical Pharmacology

Sofia Attelind[1,2], Niclas Eriksson[1,3], Mia Wadelius[1], and Pär Hallberg[1].
[1] Department of Medical Sciences, Clinical Pharmacogenomics, Uppsala University, Uppsala, Sweden
[2] Department of Drug Safety, Swedish Medical Products Agency, Uppsala, Sweden
[3] Uppsala Clinical Research Center, Uppsala University Hospital, Uppsala, Sweden

Corresponding Author: Sofia Attelind, Department of Medical Sciences, Clinical Pharmacogenomics, Uppsala University, Se-751 85 Uppsala, Sweden. e-mail: sofia.attelind@medsci.uu.se

Contents

Supplementary Fig. S1 Power calculations for GWAS using an additive genetic model 2

Supplementary Fig. S2 Principal component (PC) analysis of PC1 and PC2 for all cases........................... 3

Supplementary Fig. S3 Manhattan plot of the genome-wide association study for all cases of bleeding (n=129) vs all population controls (n=4891). 4

Supplementary Fig. S4 Manhattan plot of the genome-wide association study including cases of major or clinically relevant non-major bleeding (n=92) vs population controls matched for DOAC exposure (n=353). 5

Supplementary Table S1. Top 60 results of the genome-wide association analysis for all cases of bleeding (n=129) vs all controls (n=4891) 6

Supplementary Table S2. Top 60 results of the genome-wide association analyses of all cases (n=129) vs matched controls (n=353) 9

Supplementary Table S3. Top 60 results of the genome-wide association analyses of cases of major and clinically relevant non-major bleeding (n=92) vs matched controls (n=353) 12

Supplementary Table S4. Candidate variant results for cases of major bleeding due to Xa inhibitors vs controls matched for DOAC exposure 15

Supplementary Table S5. Candidate gene set results for all cases of bleeding (n=129) vs all controls (n=4891) 16

Supplementary Table S6. Candidate gene set results for all cases of bleeding on factor Xa inhibitors (n=115) vs all controls (n=4891) 19

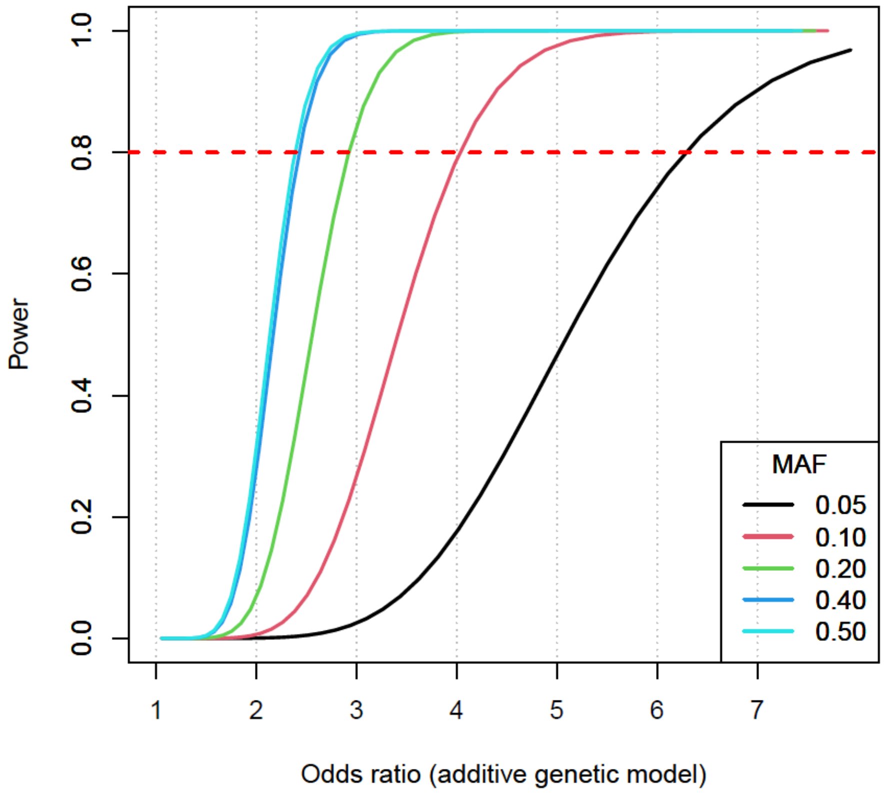

Supplementary Fig. S1 Power calculations for GWAS using an additive genetic model with 100 cases and 5000 controls, 1 million tests and assumed disease prevalence of 0.02.

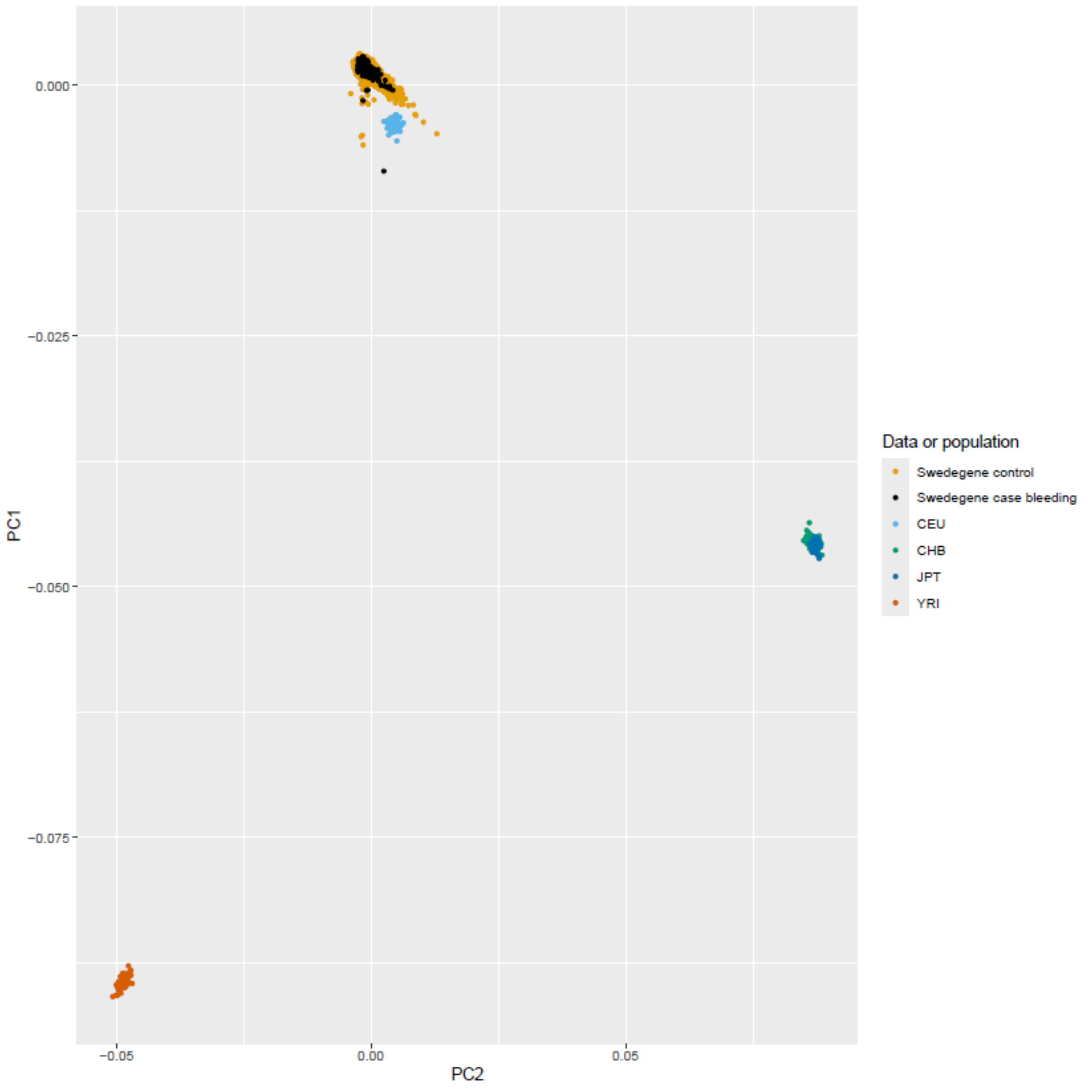

Supplementary Fig. S2 Principal component (PC) analysis of PC1 and PC2 for all cases (Swedegene case bleeding, n=129) and all controls (Swedegene control, N=4891). Comparison in HapMap with Utah residents with Northern and Western European ancestry from the CEPH collection (CEU), Han Chinese in Beijing, China (CHB), Japanese in Tokyo, Japan (JPT), and Yoruba people in Ibadan, Nigeria (YRI).

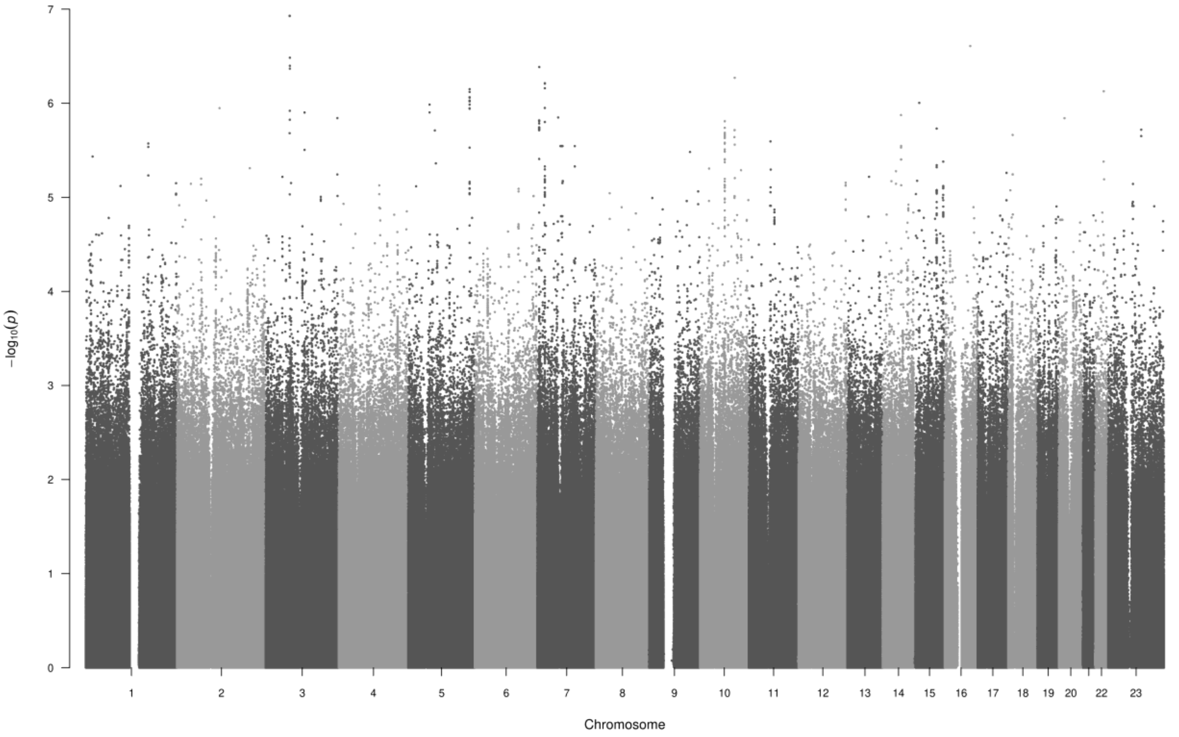

Supplementary Fig. S3 Manhattan plot of the genome-wide association study for all cases of bleeding (n=129) vs all population controls (n=4891).

Adjusted by sex and genetic principal components 1-6. No SNP passed the significance threshold $P < 5\times10^{-8}$.

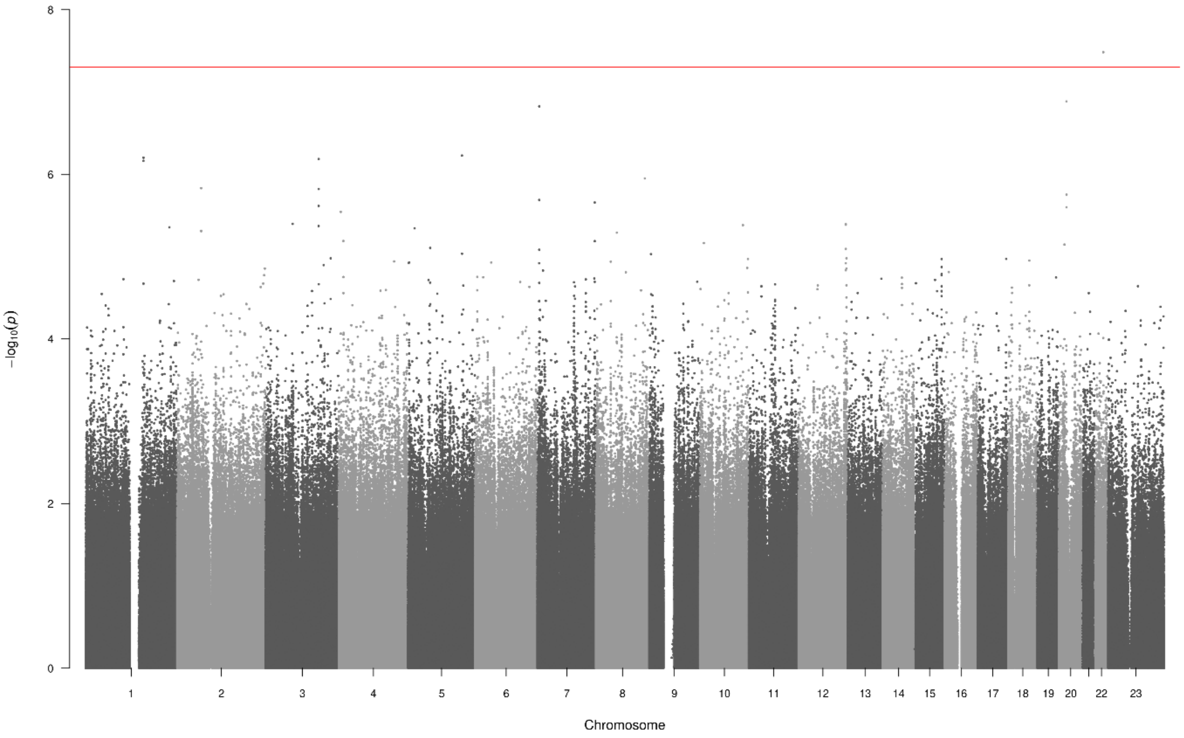

Supplementary Fig. S4 Manhattan plot of the genome-wide association study including cases of major or clinically relevant non-major bleeding (n=92) vs population controls matched for DOAC exposure (n=353).

Adjusted by sex and genetic principal components 1-6. The red line denotes the genome-wide significance level P $<5\times10^{-8}$.

Supplementary Table S1. Top 60 results of the genome-wide association analysis for all cases of bleeding (n=129) vs all controls (n=4891)

CHR	Position[a]	SNP	Alleles minor/major	MAF cases	MAF controls	N cases het/hom	N controls het/hom	OR [95% CI]	P	Gene
3	64575880	rs1176086157	A/T	0.0194	0.0003	5/0	3/0	76.0 [16.8, 342.8]	1.17822×10^{-7}	ADAMTS9 | ADAMTS9-AS1
3	64594023	rs1379550296	GC/G	0.0194	0.0003	5/0	3/0	76.0 [16.8, 342.8]	1.17822×10^{-7}	ADAMTS9
3	64656565	rs989472990	A/G	0.0194	0.0003	5/0	3/0	76.0 [16.8, 342.8]	1.17822×10^{-7}	ADAMTS9
3	65208193	rs1230434154	C/T	0.0194	0.0003	5/0	3/0	76.0 [16.8, 342.8]	1.17822×10^{-7}	
16	70001824	rs764364621	A/G	0.0194	0.0004	5/0	4/0	53.5 [14.2, 202.5]	2.4669×10^{-7}	
3	65294209	rs145286477	C/T	0.0271	0.0024	7/0	23/0	19.4 [7.9, 47.7]	3.28121×10^{-7}	
3	65316707	rs151062472	A/G	0.0271	0.0024	7/0	23/0	18.7 [7.6, 45.8]	4.00182×10^{-7}	
7	4823046	rs7800598	A/G	0.1744	0.0762	37/4	661/42	2.5 [1.8, 3.4]	4.11917×10^{-7}	RADIL
3	65297345	rs571615079	C/G	0.0271	0.0025	7/0	24/0	18.4 [7.5, 45.0]	4.29189×10^{-7}	
10	94978418	rs546704604	C/T	0.0233	0.0009	6/0	9/0	26.2 [9.1, 75.2]	5.36181×10^{-7}	CYP2C9
7	20212924	rs1859809	T/G	0.3101	0.4634	50/15	2463/1035	0.5 [0.4, 0.7]	6.12782×10^{-7}	MACC1
7	20212410	rs2190434	G/A	0.3256	0.4785	54/15	2479/1101	0.5 [0.4, 0.7]	6.18706×10^{-7}	MACC1
7	20200254	rs147764454	T/TAGATTTAAA	0.5698	0.4173	63/42	2420/831	1.9 [1.5, 2.4]	6.92872×10^{-7}	MACC1
5	166880125	rs139563295	T/C	0.1085	0.0374	22/3	350/8	3.2 [2.1, 4.8]	7.07635×10^{-7}	
22	39199202	rs1050613951	T/C	0.0194	0.0005	5/0	5/0	41.1 [11.5, 146.8]	7.46316×10^{-7}	
5	166905920	rs148147526	CCCTGG/C	0.1124	0.0400	23/3	371/10	3.1 [2.1, 4.6]	7.57815×10^{-7}	LINC01947
5	166891294	rs146888861	A/G	0.1085	0.0377	22/3	351/9	3.1 [2.1, 4.7]	8.62371×10^{-7}	
5	166891993	rs76107590	T/C	0.1085	0.0377	22/3	351/9	3.1 [2.1, 4.7]	8.62371×10^{-7}	
5	166895614	rs114434641	C/T	0.1085	0.0378	22/3	352/9	3.1 [2.1, 4.7]	8.71812×10^{-7}	
5	166886238	rs4869014	T/C	0.1085	0.0379	22/3	353/9	3.1 [2.1, 4.7]	9.35719×10^{-7}	
5	166899071	rs75831066	C/A	0.1085	0.0380	22/3	354/9	3.1 [2.1, 4.7]	9.5593×10^{-7}	
15	33013989	rs754042618	G/A	0.0116	0.0000	3/0	0/0	1986.6 [40.9, 96521.2]	9.91671×10^{-7}	FMN1
5	57756326	rs183158424	A/T	0.0310	0.0031	8/0	30/0	12.1 [5.5, 26.9]	1.03285×10^{-6}	
5	166889949	rs73800834	C/G	0.1085	0.0381	22/3	355/9	3.1 [2.1, 4.7]	1.03338×10^{-6}	

CHR	Position[a]	SNP	Alleles minor/major	MAF cases	MAF controls	N cases het/hom	N controls het/hom	OR [95% CI]	P	Gene
7	20203413	rs3114473	C/A	0.5736	0.4237	62/43	2419/863	1.9 [1.5, 2.4]	1.12291×10^{-6}	MACC1
2	115799191	rs754193660	G/A	0.0155	0.0003	4/0	3/0	82.7 [15.9, 430.5]	1.12798×10^{-6}	DPP10
5	166881864	rs4460157	C/T	0.1085	0.0383	22/3	357/9	3.1 [2.1, 4.6]	1.13146×10^{-6}	
5	166882427	rs4583907	G/A	0.1085	0.0384	22/3	358/9	3.1 [2.1, 4.6]	1.14363×10^{-6}	
3	65243000	rs545540560	C/T	0.0271	0.0028	7/0	27/0	15.4 [6.4, 36.8]	1.20247×10^{-6}	
5	57773104	rs145973246	C/T	0.0310	0.0032	8/0	31/0	11.8 [5.3, 26.0]	1.25199×10^{-6}	
3	105346157	rs532129583	A/G	0.0271	0.0019	7/0	19/0	15.3 [6.3, 37.2]	1.25456×10^{-6}	
14	70189130	rs140381706	T/C	0.0271	0.0017	7/0	17/0	15.3 [6.3, 37.5]	1.33576×10^{-6}	
7	56490073	rs773414441	T/TTTTGCCATGTTGGGG	0.0194	0.0005	5/0	5/0	35.6 [10.1, 125.8]	1.41643×10^{-6}	
7	56530648	rs185684605	T/C	0.0194	0.0005	5/0	5/0	35.6 [10.1, 125.8]	1.41643×10^{-6}	
7	56552276	rs544296436	A/T	0.0194	0.0005	5/0	5/0	35.6 [10.1, 125.8]	1.41643×10^{-6}	
7	56553616	rs764874253	T/C	0.0194	0.0005	5/0	5/0	35.6 [10.1, 125.8]	1.41643×10^{-6}	
3	194660230	rs148207521	A/T	0.0465	0.0077	12/0	75/0	6.6 [3.5, 12.4]	1.43849×10^{-6}	LSG1
20	14956418	rs151310336	G/A	0.0698	0.0181	18/0	177/0	4.4 [2.6, 7.3]	1.44155×10^{-6}	MACROD2
3	65228149	rs138608772	A/T	0.0271	0.0029	7/0	28/0	14.8 [6.2, 35.2]	1.49633×10^{-6}	
7	4789740	rs12155405	G/C	0.1124	0.0409	27/1	376/12	3.0 [2.0, 4.4]	1.5196×10^{-6}	AP5Z1
7	4790324	rs749405	T/C	0.1124	0.0410	27/1	377/12	3.0 [2.0, 4.4]	1.55117×10^{-6}	AP5Z1
7	4790417	rs907718	G/T	0.1124	0.0410	27/1	377/12	3.0 [2.0, 4.4]	1.55117×10^{-6}	AP5Z1
7	4791661	rs12154622	C/T	0.1124	0.0410	27/1	377/12	3.0 [2.0, 4.4]	1.55117×10^{-6}	AP5Z1
7	4791763	rs12154696	C/T	0.1124	0.0410	27/1	377/12	3.0 [2.0, 4.4]	1.55117×10^{-6}	AP5Z1
10	68090709	rs8181450	T/C	0.1667	0.0748	35/4	678/27	2.4 [1.7, 3.4]	1.55354×10^{-6}	
10	68091719	rs10997930	A/G	0.1667	0.0748	35/4	678/27	2.4 [1.7, 3.4]	1.55354×10^{-6}	
7	20203540	rs3095011	A/T	0.5698	0.4222	63/42	2414/858	1.9 [1.4, 2.4]	1.58289×10^{-6}	MACC1
7	4794809	rs73305400	G/A	0.1124	0.0411	27/1	378/12	2.9 [2.0, 4.4]	1.64242×10^{-6}	
7	4791430	rs12154545	G/A	0.1124	0.0413	27/1	380/12	2.9 [2.0, 4.3]	1.80244×10^{-6}	AP5Z1
7	4779713	rs148365319	C/T	0.1124	0.0412	27/1	379/12	2.9 [2.0, 4.3]	1.80315×10^{-6}	AP5Z1
7	4779757	rs7798106	T/C	0.1124	0.0412	27/1	379/12	2.9 [2.0, 4.3]	1.80315×10^{-6}	AP5Z1
7	4783256	rs77086996	C/T	0.1124	0.0413	27/1	380/12	2.9 [2.0, 4.3]	1.80912×10^{-6}	AP5Z1

CHR	Position[a]	SNP	Alleles minor/major	MAF cases	MAF controls	N cases het/hom	N controls het/hom	OR [95% CI]	P	Gene
10	68004832	rs10823124	A/C	0.1628	0.0732	34/4	668/24	2.5 [1.8, 3.5]	1.82229×10^{-6}	HERC4
7	4777037	rs80200305	A/C	0.1124	0.0413	27/1	380/12	2.9 [2.0, 4.3]	1.82387×10^{-6}	AP5Z1
15	80552795	rs3924894	T/C	0.5465	0.3981	59/41	2418/738	1.9 [1.4, 2.4]	1.85992×10^{-6}	ARNT2
15	80552796	rs3924893	C/T	0.5465	0.3981	59/41	2418/738	1.9 [1.4, 2.4]	1.85992×10^{-6}	ARNT2
7	4779479	rs191242271	C/T	0.1124	0.0412	27/1	379/12	2.9 [2.0, 4.3]	1.86227×10^{-6}	AP5Z1
7	4780129	rs7781254	G/A	0.1124	0.0413	27/1	380/12	2.9 [2.0, 4.3]	1.86553×10^{-6}	AP5Z1
7	4777829	rs7810433	C/T	0.1124	0.0413	27/1	380/12	2.9 [2.0, 4.3]	1.89577×10^{-6}	AP5Z1
7	4778321	rs17135113	T/G	0.1124	0.0413	27/1	380/12	2.9 [2.0, 4.3]	1.89577×10^{-6}	AP5Z1

CHR, chromosome; SNP, single nucleotide polymorphism; MAF, minor allele frequency; N is the number of non-missing values; OR, odds ratio with lower and upper 95% confidence interval (CI).

* The P-value for significance was set to the conventional genome-wide significance threshold $P < 5\times10^{-8}$. Total number of SNPs with results 24049323. More data provided in Table SX in the Online Resource.

Supplementary Table S2. Top 60 results of the genome-wide association analyses of all cases (n=129) vs matched controls (n=353)

CHR	Position[a]	SNP	Alleles minor/major	MAF cases	MAF controls	N cases/controls	N cases het/hom	N controls het/hom	OR [95% CI]	P	Gene
22	38087164	rs142001534	A/G	0.0659	0.0071	129/353	17/0	5/0	12.1 [4.3, 34.2]	4.66015×10^{-8}*	BAIAP2L2
7	4823046	rs7800598	A/G	0.1744	0.0652	129/353	37/4	46/0	3.3 [2.0, 5.2]	3.12994×10^{-7}	RADIL
7	4779479	rs191242271	C/T	0.1124	0.0297	129/353	27/1	21/0	4.5 [2.5, 8.4]	3.87561×10^{-7}	AP5Z1
12	128850145	rs11059972	T/C	0.5271	0.3470	129/353	62/37	163/41	2.1 [1.6, 2.9]	3.95204×10^{-7}	
12	128850269	rs11059973	T/C	0.5271	0.3470	129/353	62/37	163/41	2.1 [1.6, 2.9]	3.95204×10^{-7}	
12	128854632	rs12312389	T/G	0.5271	0.3484	129/353	62/37	166/40	2.1 [1.6, 2.9]	4.18103×10^{-7}	GLT1D1
7	4775504	rs35684868	A/C	0.1124	0.0312	129/353	27/1	22/0	4.4 [2.4, 8.0]	5.75023×10^{-7}	
7	4777037	rs80200305	A/C	0.1124	0.0312	129/353	27/1	22/0	4.4 [2.4, 8.0]	5.75023×10^{-7}	AP5Z1
7	4777829	rs7810433	C/T	0.1124	0.0312	129/353	27/1	22/0	4.4 [2.4, 8.0]	5.75023×10^{-7}	AP5Z1
7	4778321	rs17135113	T/G	0.1124	0.0312	129/353	27/1	22/0	4.4 [2.4, 8.0]	5.75023×10^{-7}	AP5Z1
7	4779713	rs148365319	C/T	0.1124	0.0312	129/353	27/1	22/0	4.4 [2.4, 8.0]	5.75023×10^{-7}	AP5Z1
7	4779757	rs7798106	T/C	0.1124	0.0312	129/353	27/1	22/0	4.4 [2.4, 8.0]	5.75023×10^{-7}	AP5Z1
7	4780129	rs7781254	G/A	0.1124	0.0312	129/353	27/1	22/0	4.4 [2.4, 8.0]	5.75023×10^{-7}	AP5Z1
7	4783256	rs77086996	C/T	0.1124	0.0312	129/353	27/1	22/0	4.4 [2.4, 8.0]	5.75023×10^{-7}	AP5Z1
7	4790324	rs749405	T/C	0.1124	0.0312	129/353	27/1	22/0	4.4 [2.4, 8.0]	5.75023×10^{-7}	AP5Z1
7	4790417	rs907718	G/T	0.1124	0.0312	129/353	27/1	22/0	4.4 [2.4, 8.0]	5.75023×10^{-7}	AP5Z1
7	4791430	rs12154545	G/A	0.1124	0.0312	129/353	27/1	22/0	4.4 [2.4, 8.0]	5.75023×10^{-7}	AP5Z1
7	4791661	rs12154622	C/T	0.1124	0.0312	129/353	27/1	22/0	4.4 [2.4, 8.0]	5.75023×10^{-7}	AP5Z1
7	4791763	rs12154696	C/T	0.1124	0.0312	129/353	27/1	22/0	4.4 [2.4, 8.0]	5.75023×10^{-7}	AP5Z1
12	128835633	rs2398437	C/T	0.5349	0.3612	129/353	60/39	173/41	2.1 [1.6, 2.9]	6.62841×10^{-7}	
8	133584023	rs79710501	A/G	0.0388	0.0000	129/353	10/0	0/0	58.0 [3.3, 1019.0]	7.58758×10^{-7}	
8	133586915	rs113400554	T/C	0.0388	0.0000	129/353	10/0	0/0	58.0 [3.3, 1019.0]	7.58758×10^{-7}	
7	4778660	rs6979798	A/T	0.1124	0.0326	129/353	27/1	23/0	4.2 [2.3, 7.5]	1.1489×10^{-6}	AP5Z1
7	4779806	rs540584247	A/AT	0.1124	0.0326	129/353	27/1	23/0	4.2 [2.3, 7.5]	1.1489×10^{-6}	AP5Z1
7	4780792	rs76043497	C/T	0.1124	0.0326	129/353	27/1	23/0	4.2 [2.3, 7.5]	1.1489×10^{-6}	AP5Z1

CHR	Position[a]	SNP	Alleles minor/major	MAF cases	MAF controls	N cases/controls	N cases het/hom	N controls het/hom	OR [95% CI]	P	Gene
7	4789740	rs12155405	G/C	0.1124	0.0326	129/353	27/1	23/0	4.2 [2.3, 7.5]	1.1489×10^{-6}	AP5Z1
7	4794809	rs73305400	G/A	0.1124	0.0326	129/353	27/1	23/0	4.2 [2.3, 7.5]	1.1489×10^{-6}	
12	128827676	rs6486731	A/G	0.5271	0.3598	129/353	62/37	172/41	2.1 [1.5, 2.8]	1.31176×10^{-6}	
12	128828272	rs6486732	G/C	0.5271	0.3598	129/353	62/37	172/41	2.1 [1.5, 2.8]	1.31176×10^{-6}	
5	17013647	rs147513370	A/G	0.0504	0.0042	129/353	13/0	3/0	12.4 [3.6, 42.7]	1.32468×10^{-6}	
4	6011659	rs187018832	A/C	0.0349	0.0000	129/353	9/0	0/0	57.0 [2.8, 1158.0]	1.35126×10^{-6}	C4orf50
7	4792338	rs11976063	C/T	0.1085	0.0312	129/353	26/1	22/0	4.2 [2.3, 7.7]	0.000001458	AP5Z1
1	103171242	rs116708605	C/T	0.0504	0.0057	129/353	13/0	4/0	11.5 [3.6, 36.9]	1.68555×10^{-6}	
1	103199864	rs549040518	G/A	0.0504	0.0057	129/353	13/0	4/0	11.5 [3.6, 36.9]	1.68555×10^{-6}	
1	103249699	rs201307070	C/CTA	0.0504	0.0057	129/353	13/0	4/0	11.5 [3.6, 36.9]	1.68555×10^{-6}	
1	103266615	rs201397354	AT/A	0.0504	0.0057	129/353	13/0	4/0	11.5 [3.6, 36.9]	1.68555×10^{-6}	
12	128850309	rs11059974	G/A	0.5814	0.4079	129/353	62/44	172/58	2.0 [1.5, 2.7]	1.90391×10^{-6}	
1	157642971	rs148139317	G/A	0.0426	0.0014	129/353	9/1	1/0	20.4 [3.3, 124.1]	2.27507×10^{-6}	
1	157813144	rs78105988	A/G	0.0426	0.0014	129/353	9/1	1/0	20.4 [3.3, 124.1]	2.27507×10^{-6}	FCRL1
1	157818262	rs76005673	C/T	0.0426	0.0014	129/353	9/1	1/0	20.4 [3.3, 124.1]	2.27507×10^{-6}	FCRL1
3	149553300	rs11716804	C/T	0.2287	0.3881	129/353	43/8	180/47	0.4 [0.3, 0.6]	2.36466×10^{-6}	WWTR1
3	149557874	rs6787616	A/G	0.2287	0.3881	129/353	43/8	180/47	0.4 [0.3, 0.6]	2.36466×10^{-6}	WWTR1
3	149558925	rs62269314	A/G	0.2287	0.3881	129/353	43/8	180/47	0.4 [0.3, 0.6]	2.36466×10^{-6}	WWTR1
1	157618301	rs146783641	C/G	0.0388	0.0014	129/353	10/0	1/0	22.2 [3.6, 136.1]	2.42823×10^{-6}	
11	34297911	rs78627928	A/G	0.1744	0.0680	129/353	37/4	44/2	2.8 [1.8, 4.4]	2.48851×10^{-6}	ABTB2
11	34292629	rs881390	C/T	0.1744	0.0680	129/353	37/4	44/2	2.8 [1.8, 4.4]	2.50194×10^{-6}	ABTB2
3	143961036	rs73159881	T/A	0.0659	0.0127	129/353	15/1	9/0	6.8 [2.8, 16.3]	2.86819×10^{-6}	
11	59595947	rs557374538	C/A	0.0543	0.0071	129/353	14/0	5/0	8.8 [3.1, 25.0]	3.23224×10^{-6}	OSBP
20	14956418	rs151310336	G/A	0.0698	0.0127	129/353	18/0	9/0	6.2 [2.7, 14.3]	3.44359×10^{-6}	MACROD2
11	34293891	rs4756131	G/A	0.1744	0.0694	129/353	37/4	45/2	2.8 [1.8, 4.3]	3.58647×10^{-6}	ABTB2
11	34294889	rs117247679	T/A	0.1744	0.0694	129/353	37/4	45/2	2.8 [1.8, 4.3]	3.58647×10^{-6}	ABTB2
1	159711078	rs2808630	C/T	0.1899	0.3385	129/353	41/4	159/40	0.4 [0.3, 0.6]	3.69486×10^{-6}	

CHR	Position[a]	SNP	Alleles minor/major	MAF cases	MAF controls	N cases/controls	N cases het/hom	N controls het/hom	OR [95% CI]	P	Gene
3	143864290	rs73159833	C/T	0.0659	0.0127	129/353	15/1	9/0	6.6 [2.7, 15.7]	4.12414×10^{-6}	
4	119348788	rs140829909	G/A	0.0310	0.0000	129/353	8/0	0/0	52.8 [2.5, 1093.8]	4.68794×10^{-6}	KLHL2P1
1	62038283	rs6682768	G/A	0.0426	0.0028	129/353	11/0	2/0	15.1 [3.5, 64.5]	4.87873×10^{-6}	PATJ
1	62038389	rs6658506	G/C	0.0426	0.0028	129/353	11/0	2/0	15.1 [3.5, 64.5]	4.87873×10^{-6}	PATJ
1	62041927	rs149583642	A/C	0.0426	0.0028	129/353	11/0	2/0	15.1 [3.5, 64.5]	4.87873×10^{-6}	PATJ
1	62042101	rs60496532	T/C	0.0426	0.0028	129/353	11/0	2/0	15.1 [3.5, 64.5]	4.87873×10^{-6}	PATJ
1	62042316	rs7526702	T/C	0.0426	0.0028	129/353	11/0	2/0	15.1 [3.5, 64.5]	4.87873×10^{-6}	PATJ
1	62042447	rs7551434	G/A	0.0426	0.0028	129/353	11/0	2/0	15.1 [3.5, 64.5]	4.87873×10^{-6}	PATJ

CHR, chromosome; SNP, single nucleotide polymorphism; MAF, minor allele frequency; N is the number of non-missing values; OR, odds ratio with lower and upper 95% confidence interval (CI).

* The P-value for significance was set to the conventional genome-wide significance threshold $P < 5\times10^{-8}$. Total number of SNPs with results 15688153.

Supplementary Table S3. Top 60 results of the genome-wide association analyses of cases of major and clinically relevant non-major bleeding (n=92) vs matched controls (n=353)

CHR	Position[a]	SNP	Alleles minor/major	MAF cases	MAF controls	N cases/controls	N cases het/hom	N controls het/hom	BETA	SE	OR [95% CI]	P	Gene
22	38087164	rs142001534	A/G	0.0761	0.0071	92/353	14/0	5/0	2.690	0.544	14.7 [5.1, 42.8]	3.28301e-08	BAIAP2L2
20	20524333	rs56098181	G/A	0.1630	0.0496	92/353	28/1	35/0	1.512	0.286	4.5 [2.6, 7.9]	1.30273e-07	RALGAPA2
7	4823046	rs7800598	A/G	0.1957	0.0652	92/353	30/3	46/0	1.353	0.260	3.9 [2.3, 6.4]	1.49555e-07	RADIL
5	146087250	rs114735777	G/A	0.0761	0.0099	92/353	14/0	7/0	2.275	0.487	9.7 [3.7, 25.3]	5.91162e-07	PLAC8L1
1	157642971	rs148139317	G/A	0.0543	0.0014	92/353	8/1	1/0	3.294	0.942	26.9 [4.2, 170.9]	6.29349e-07	
1	157813144	rs78105988	A/G	0.0543	0.0014	92/353	8/1	1/0	3.294	0.942	26.9 [4.2, 170.9]	6.29349e-07	FCRL1
1	157818262	rs76005673	C/T	0.0543	0.0014	92/353	8/1	1/0	3.294	0.942	26.9 [4.2, 170.9]	6.29349e-07	FCRL1
3	143864290	rs73159833	C/T	0.0870	0.0127	92/353	14/1	9/0	2.100	0.450	8.2 [3.4, 19.7]	6.52315e-07	
1	157618301	rs146783641	C/G	0.0489	0.0014	92/353	9/0	1/0	3.394	0.941	29.8 [4.7, 188.4]	6.86324e-07	
8	133584023	rs79710501	A/G	0.0435	0.0000	92/353	8/0	0/0	4.203	1.544	66.9 [3.2, 1379.9]	1.12214e-06	
8	133586915	rs113400554	T/C	0.0435	0.0000	92/353	8/0	0/0	4.203	1.544	66.9 [3.2, 1379.9]	1.12214e-06	
2	65948658	rs147840322	G/A	0.0489	0.0028	92/353	9/0	2/0	3.064	0.780	21.4 [4.6, 98.7]	1.47414e-06	LINC02934
3	143961036	rs73159881	T/A	0.0815	0.0127	92/353	13/1	9/0	2.065	0.458	7.9 [3.2, 19.3]	1.50726e-06	
20	20589160	rs56006287	C/A	0.1576	0.0552	92/353	27/1	39/0	1.344	0.281	3.8 [2.2, 6.7]	1.76627e-06	RALGAPA2
7	4827306	rs113000375	A/G	0.0543	0.0042	92/353	10/0	3/0	2.702	0.661	14.9 [4.1, 54.4]	2.0543e-06	RADIL
7	156122914	rs77471862	A/G	0.0870	0.0184	92/353	14/1	13/0	1.854	0.397	6.4 [2.9, 13.9]	2.19823e-06	
3	143865278	rs73159834	C/G	0.0761	0.0113	92/353	12/1	8/0	2.107	0.480	8.2 [3.2, 21.1]	2.42412e-06	
20	20668772	rs56084826	G/A	0.1576	0.0581	92/353	27/1	41/0	1.322	0.281	3.8 [2.2, 6.5]	2.51445e-06	RALGAPA2
4	6011659	rs187018832	A/C	0.0380	0.0000	92/353	7/0	0/0	4.184	1.577	65.7 [3.0, 1445.2]	2.86227e-06	C4orf50
3	72771630	rs115605346	G/A	0.0435	0.0028	92/353	8/0	2/0	3.049	0.800	21.1 [4.4, 101.2]	3.99644e-06	SHQ1
12	128850145	rs11059972	T/C	0.5326	0.3470	92/353	46/26	163/41	0.783	0.175	2.2 [1.6, 3.1]	4.0033e-06	
12	128850269	rs11059973	T/C	0.5326	0.3470	92/353	46/26	163/41	0.783	0.175	2.2 [1.6, 3.1]	4.0033e-06	

CHR	Position[a]	SNP	Alleles minor/major	MAF cases	MAF controls	N cases/controls	N cases het/hom	N controls het/hom	BETA	SE	OR [95% CI]	P	Gene
12	128854632	rs12312389	T/G	0.5326	0.3484	92/353	46/26	166/40	0.787	0.176	2.2 [1.6, 3.1]	4.10689e-06	GLT1D1
10	117485383	rs117169231	A/G	0.1250	0.0354	92/353	23/0	25/0	1.475	0.321	4.4 [2.3, 8.2]	4.14653e-06	EMX2OS
3	143815587	rs56124843	T/C	0.0761	0.0127	92/353	12/1	9/0	2.020	0.468	7.5 [3.0, 18.8]	4.24773e-06	SLC9A9
1	228193817	rs12120621	T/C	0.3152	0.1657	92/353	42/8	95/11	0.888	0.197	2.4 [1.7, 3.6]	4.4042e-06	
5	17013647	rs147513370	A/G	0.0543	0.0042	92/353	10/0	3/0	2.593	0.656	13.4 [3.7, 48.4]	4.52773e-06	
2	65830282	rs184270304	T/A	0.0380	0.0014	92/353	7/0	1/0	3.506	1.029	33.3 [4.4, 250.3]	4.86905e-06	LINC02934
2	65938238	rs140920272	G/A	0.0489	0.0042	92/353	9/0	3/0	2.728	0.686	15.3 [4.0, 58.8]	4.91287e-06	LINC02934
8	57267891	rs145096932	A/C	0.0435	0.0014	92/353	8/0	1/0	3.189	0.943	24.3 [3.8, 154.1]	5.1168e-06	
4	13128266	rs148667386	C/G	0.0435	0.0014	92/353	8/0	1/0	3.179	0.941	24.0 [3.8, 151.9]	6.45858e-06	
7	156084426	rs113966720	A/G	0.0924	0.0255	92/353	15/1	18/0	1.688	0.375	5.4 [2.6, 11.3]	6.47517e-06	
10	11007191	rs140318622	A/G	0.0652	0.0099	92/353	12/0	7/0	2.138	0.504	8.5 [3.2, 22.8]	6.85227e-06	
20	14956418	rs151310336	G/A	0.0761	0.0127	92/353	14/0	9/0	1.920	0.447	6.8 [2.8, 16.4]	7.1381e-06	MACROD2
5	59520360	rs141916639	C/A	0.0543	0.0042	92/353	10/0	3/0	2.514	0.654	12.4 [3.4, 44.5]	7.84972e-06	PDE4D
12	128835633	rs2398437	C/T	0.5380	0.3612	92/353	45/27	173/41	0.769	0.177	2.2 [1.5, 3.1]	8.07457e-06	
7	4779479	rs191242271	C/T	0.1087	0.0297	92/353	20/0	21/0	1.514	0.341	4.5 [2.3, 8.9]	8.2589e-06	AP5Z1
5	146221127	rs115605398	A/G	0.1033	0.0269	92/353	17/1	19/0	1.528	0.352	4.6 [2.3, 9.2]	9.22572e-06	RBM27
9	4973739	rs76705823	T/C	0.0435	0.0014	92/353	8/0	1/0	3.139	0.948	23.1 [3.6, 148.1]	9.35342e-06	
9	5063893	rs147711979	G/C	0.0435	0.0014	92/353	8/0	1/0	3.139	0.948	23.1 [3.6, 148.1]	9.35342e-06	JAK2
9	5129980	rs145092116	C/T	0.0435	0.0014	92/353	8/0	1/0	3.139	0.948	23.1 [3.6, 148.1]	9.35342e-06	
9	5185130	rs569111077	T/TA	0.0435	0.0014	92/353	8/0	1/0	3.139	0.948	23.1 [3.6, 148.1]	9.35342e-06	INSL6
12	130906817	rs74242888	C/T	0.2228	0.0977	92/353	37/2	63/3	1.021	0.236	2.8 [1.7, 4.4]	1.04569e-05	
12	130910430	rs76762368	T/A	0.2228	0.0977	92/353	37/2	63/3	1.021	0.236	2.8 [1.7, 4.4]	1.04569e-05	
3	176668979	rs76569731	T/C	0.1413	0.0496	92/353	26/0	35/0	1.302	0.295	3.7 [2.1, 6.6]	1.04856e-05	LINC01208
17	78119940	rs187952596	G/A	0.0707	0.0113	92/353	13/0	8/0	1.971	0.470	7.2 [2.9, 18.0]	1.06342e-05	TMC6

CHR	Position[a]	SNP	Alleles minor/major	MAF cases	MAF controls	N cases/controls	N cases het/hom	N controls het/hom	BETA	SE	OR [95% CI]	P	Gene
10	131015919	rs112113847	A/G	0.1413	0.0482	92/353	22/2	34/0	1.254	0.289	3.5 [2.0, 6.2]	1.06837e-05	
15	94004704	rs58410675	T/C	0.2663	0.1303	92/353	37/6	78/7	0.902	0.208	2.5 [1.6, 3.7]	1.06948e-05	LINC01581
18	57643740	rs72942250	T/G	0.1304	0.0397	92/353	22/1	28/0	1.344	0.309	3.8 [2.1, 7.0]	1.11752e-05	ATP8B1-AS1
18	57645003	rs17759167	C/A	0.1304	0.0397	92/353	22/1	28/0	1.344	0.309	3.8 [2.1, 7.0]	1.11752e-05	ATP8B1-AS1
18	57645673	rs77017375	G/C	0.1304	0.0397	92/353	22/1	28/0	1.344	0.309	3.8 [2.1, 7.0]	1.11752e-05	ATP8B1-AS1
18	57648205	rs11152024	T/C	0.1304	0.0397	92/353	22/1	28/0	1.344	0.309	3.8 [2.1, 7.0]	1.11752e-05	ATP8B1-AS1 | ATP8B1
18	57649962	rs3817352	T/C	0.1304	0.0397	92/353	22/1	28/0	1.344	0.309	3.8 [2.1, 7.0]	1.11752e-05	ATP8B1-AS1 | ATP8B1
18	57652212	rs17759233	A/G	0.1304	0.0397	92/353	22/1	28/0	1.344	0.309	3.8 [2.1, 7.0]	1.11752e-05	ATP8B1-AS1 | ATP8B1
18	57653844	rs72942282	G/A	0.1304	0.0397	92/353	22/1	28/0	1.344	0.309	3.8 [2.1, 7.0]	1.11752e-05	ATP8B1-AS1 | ATP8B1
18	57654358	rs145780137	A/G	0.1304	0.0397	92/353	22/1	28/0	1.344	0.309	3.8 [2.1, 7.0]	1.11752e-05	ATP8B1-AS1 | ATP8B1
18	57654891	rs112028430	T/C	0.1304	0.0397	92/353	22/1	28/0	1.344	0.309	3.8 [2.1, 7.0]	1.11752e-05	ATP8B1-AS1 | ATP8B1
18	57657967	rs17832328	A/G	0.1304	0.0397	92/353	22/1	28/0	1.344	0.309	3.8 [2.1, 7.0]	1.11752e-05	ATP8B1-AS1 | ATP8B1
18	57658849	rs72942297	C/A	0.1304	0.0397	92/353	22/1	28/0	1.344	0.309	3.8 [2.1, 7.0]	1.11752e-05	ATP8B1-AS1 | ATP8B1
18	57659538	rs17832346	A/G	0.1304	0.0397	92/353	22/1	28/0	1.344	0.309	3.8 [2.1, 7.0]	1.11752e-05	ATP8B1-AS1 | ATP8B1

CHR, chromosome; SNP, single nucleotide polymorphism; MAF, minor allele frequency; N is the number of non-missing values; BETA, the beta coefficient is the degree of change in the outcome variable for every unit of change in the predictor variable; SE, standard error; OR, odds ratio with lower and upper 95% confidence interval (CI).

[a] Position base pair: all results are reported in accordance with the Genome Reference Consortium human assembly GRCh38. All results are presented on the forward strand. Total number of SNPs with results 15426582.

Supplementary Table S4. Candidate variant results for cases of major bleeding due to Xa inhibitors vs controls matched for DOAC exposure

CHR	Position[a]	SNP	Alleles minor/major	MAF cases	MAF controls	N cases/ controls	OR [95% CI]	P	Gene
7	99672916	rs776746[b]	T/C	0.207	0.058	41/353	4.60 [2.31, 9.16]	2.458×10^{-5}*	CYP3A5
7	99784473	rs2740574	C/T	0.085	0.034	41/353	2.56 [1.06, 6.16]	0.0397821	CYP3A4
4	88139962	rs2231137	T/C	0.012	0.059	41/353	0.40 [0.15, 1.13]	0.0826683	ABCG2
7	87509329	rs1045642	G/A	0.476	0.405	41/353	1.32 [0.84, 2.09]	0.2354221	ABCB1
4	88131171	rs2231142	T/G	0.122	0.086	41/353	1.51 [0.67, 3.39]	0.3165822	ABCG2
16	55827882	rs8192935	A/G	0.293	0.336	41/353	0.80 [0.47, 1.36]	0.4040255	CES1
7	87550285	rs1128503	A/G	0.415	0.456	41/353	0.88 [0.56, 1.38]	0.5753271	ABCB1
16	55810697	rs2244613	G/T	0.159	0.176	41/353	0.89 [0.46, 1.70]	0.7183165	CES1
7	99768693	rs35599367	A/G	0.037	0.034	41/353	0.96 [0.29, 3.18]	0.9477384	CYP3A4

CHR, chromosome; SNP, single nucleotide polymorphism; MAF, minor allele frequency; N is the number of non-missing values; OR, odds ratio with lower and upper 95% confidence interval (CI).

* The P-value for significance was set to 0.00556, according to Bonferroni adjustment for the number of tested SNPs (n=9).

[a] All results are reported in accordance with the Genome Reference Consortium human assembly GRCh38.

[b] **Please note that CYP3A5 is read on the reverse strand, i.e. rs776746 T/C is A/G in the gene's sense strand.** All results are reported on the forward strand.

Supplementary Table S5. Candidate gene set results for all cases of bleeding (n=129) vs all controls (n=4891)

Region	CHR	max MAF	P value SKAT-O	P value Burden	P value SKAT	BETA Burden	SE Burden	MAC case	MAC control	Number rare	Number ultra rare
VWF	12	0.001	0.00296*	0.00296	0.00296	0.10360	0.0349	5	41	0	18
VWF	12	0.01	0.01726	0.20006	0.00962	0.02289	0.0179	9	206	6	18
VWF	12	0.4	0.17484	0.13123	0.12328	0.01577	0.0105	361	13470	19	18
VWF	12	0.2	0.17484	0.13123	0.12328	0.01577	0.0105	167	6199	17	18
VWF	12	0.1	0.17713	0.13455	0.12192	0.01589	0.0106	101	3816	15	18
FGG	4	0.001	0.05493	0.05493	0.05493	0.16716	0.0871	1	3	0	3
FGG	4	0.1	0.4424	0.75552	0.31529	-0.01092	0.0351	1	54	2	3
FGG	4	0.2	0.4424	0.75552	0.31529	-0.01092	0.0351	1	54	2	3
FGG	4	0.01	0.4424	0.75552	0.31529	-0.01092	0.0351	1	54	2	3
FGG	4	0.4	0.4424	0.75552	0.31529	-0.01092	0.0351	1	54	2	3
FGB	4	0.01	0.07477	0.46268	0.04654	0.01947	0.0265	4	92	4	0
FGB	4	0.1	0.07477	0.46268	0.04654	0.01947	0.0265	4	92	4	0
FGB	4	0.2	0.07481	0.45243	0.04656	0.01989	0.0265	59	1979	5	0
FGB	4	0.4	0.07481	0.45243	0.04656	0.01989	0.0265	59	1979	5	0
ABCB1	7	0.4	0.08621	0.05021	0.49426	-0.03520	0.0180	40	1914	6	11
ABCB1	7	0.2	0.08621	0.05021	0.49426	-0.03520	0.0180	40	1914	6	11
ABCB1	7	0.1	0.09492	0.05563	0.49157	-0.03465	0.0181	8	582	5	11
ABCB1	7	0.01	0.21277	0.13518	0.46673	-0.04461	0.0299	0	87	3	11
ABCB1	7	0.001	0.26291	0.26291	0.26291	-0.04634	0.0414	0	45	0	11
CES2	16	0.001	0.1891	0.1891	0.1891	0.10235	0.0779	1	6	0	3
CES2	16	0.4	0.54764	0.39462	0.44507	0.03406	0.0400	2	36	2	3
CES2	16	0.01	0.54764	0.39462	0.44507	0.03406	0.0400	2	36	2	3
CES2	16	0.1	0.54764	0.39462	0.44507	0.03406	0.0400	2	36	2	3
CES2	16	0.2	0.54764	0.39462	0.44507	0.03406	0.0400	2	36	2	3
CES1	16	0.01	0.19841	0.20141	0.16424	0.04254	0.0333	4	80	1	2
CES1	16	0.1	0.57657	0.55072	0.51011	0.01650	0.0277	16	497	3	2
CES1	16	0.4	0.57657	0.55072	0.51011	0.01650	0.0277	16	497	3	2

Region	CHR	max MAF	P value SKAT-O	P value Burden	P value SKAT	BETA Burden	SE Burden	MAC case	MAC control	Number rare	Number ultra rare
CES1	16	0.2	0.57657	0.55072	0.51011	0.01650	0.0277	16	497	3	2
CES1	16	0.001	0.73732	0.73732	0.73732	-0.04196	0.1251	0	3	0	2
ABCG2	4	0.01	0.22354	0.296	0.14121	0.02694	0.0258	5	120	3	4
ABCG2	4	0.2	0.26049	0.40943	0.16352	0.01673	0.0203	49	1504	5	4
ABCG2	4	0.4	0.26049	0.40943	0.16352	0.01673	0.0203	49	1504	5	4
ABCG2	4	0.1	0.26049	0.40943	0.16352	0.01673	0.0203	49	1504	5	4
ABCG2	4	0.001	0.61789	0.61789	0.61789	-0.04216	0.0845	0	9	0	4
CYP3A5	7	0.001	0.29033	0.29033	0.29033	0.07320	0.0692	1	13	0	4
CYP3A5	7	0.1	0.63931	0.45685	0.71454	0.01376	0.0185	9	269	4	4
CYP3A5	7	0.4	0.63931	0.45685	0.71454	0.01376	0.0185	9	269	4	4
CYP3A5	7	0.2	0.63931	0.45685	0.71454	0.01376	0.0185	9	269	4	4
CYP3A5	7	0.01	0.76825	0.86454	0.60796	0.00546	0.0320	2	66	2	4
SERPINE1	7	0.4	0.38283	0.46717	0.27565	-0.02067	0.0284	34	1070	2	3
SERPINE1	7	0.2	0.38283	0.46717	0.27565	-0.02067	0.0284	34	1070	2	3
SERPINE1	7	0.1	0.38283	0.46717	0.27565	-0.02067	0.0284	34	1070	2	3
SERPINE1	7	0.001	0.60571	0.60571	0.60571	-0.04230	0.0819	0	10	0	3
SERPINE1	7	0.01	0.60571	0.60571	0.60571	-0.04230	0.0819	0	10	0	3
SERPINF2	17	0.4	0.39584	0.29121	0.57845	-0.04363	0.0413	164	6526	4	7
SERPINF2	17	0.01	0.39865	0.29373	0.57747	-0.04348	0.0414	0	41	1	7
SERPINF2	17	0.1	0.39865	0.29373	0.57747	-0.04348	0.0414	0	41	1	7
SERPINF2	17	0.2	0.40171	0.29609	0.57834	-0.04320	0.0413	51	1916	2	7
SERPINF2	17	0.001	0.45937	0.45937	0.45937	-0.04358	0.0589	0	22	0	7
SULT1A1	16	0.01	0.42457	0.42457	0.42457	0.04956	0.0621	1	15	0	3
SULT1A1	16	0.1	0.42457	0.42457	0.42457	0.04956	0.0621	1	15	0	3
SULT1A1	16	0.4	0.42457	0.42457	0.42457	0.04956	0.0621	1	15	0	3
SULT1A1	16	0.2	0.42457	0.42457	0.42457	0.04956	0.0621	1	15	0	3
SULT1A1	16	0.001	0.42457	0.42457	0.42457	0.04956	0.0621	1	15	0	3
GGCX	2	0.01	0.44393	0.44393	0.44393	-0.04356	0.0569	0	20	0	7

Region	CHR	max MAF	P value SKAT-O	P value Burden	P value SKAT	BETA Burden	SE Burden	MAC case	MAC control	Number rare	Number ultra rare
GGCX	2	0.001	0.44393	0.44393	0.44393	-0.04356	0.0569	0	20	0	7
GGCX	2	0.1	0.44393	0.44393	0.44393	-0.04356	0.0569	0	20	0	7
GGCX	2	0.2	0.44393	0.44393	0.44393	-0.04356	0.0569	0	20	0	7
GGCX	2	0.4	0.44415	0.44409	0.44393	-0.04354	0.0569	87	3223	1	7
THBD	20	0.4	0.48714	0.36584	0.58959	-0.04362	0.0482	53	2269	2	2
THBD	20	0.1	0.49547	0.373	0.58954	-0.04299	0.0483	0	29	1	2
THBD	20	0.01	0.49547	0.373	0.58954	-0.04299	0.0483	0	29	1	2
THBD	20	0.2	0.49547	0.373	0.58954	-0.04299	0.0483	0	29	1	2
THBD	20	0.001	0.63578	0.63578	0.63578	-0.04208	0.0889	0	9	0	2
F12	5	0.001	0.57424	0.57424	0.57424	-0.04243	0.0755	0	11	0	3
F12	5	0.4	0.75562	0.59644	0.74889	-0.01757	0.0332	1	60	2	3
F12	5	0.1	0.75562	0.59644	0.74889	-0.01757	0.0332	1	60	2	3
F12	5	0.2	0.75562	0.59644	0.74889	-0.01757	0.0332	1	60	2	3
F12	5	0.01	0.75562	0.59644	0.74889	-0.01757	0.0332	1	60	2	3
CYP3A4	7	0.001	0.59859	0.59859	0.59859	-0.04223	0.0802	0	10	0	3
CYP3A4	7	0.2	0.71641	0.56522	0.65443	-0.01547	0.0269	2	125	2	3
CYP3A4	7	0.4	0.71641	0.56522	0.65443	-0.01547	0.0269	2	125	2	3
CYP3A4	7	0.01	0.71641	0.56522	0.65443	-0.01547	0.0269	2	125	2	3
CYP3A4	7	0.1	0.71641	0.56522	0.65443	-0.01547	0.0269	2	125	2	3
FGA	4	0.4	0.70603	0.57269	0.85576	-0.04272	0.0757	63	2627	2	2
FGA	4	0.1	0.71039	0.57731	0.85574	-0.04220	0.0757	0	18	1	2
FGA	4	0.2	0.71039	0.57731	0.85574	-0.04220	0.0757	0	18	1	2
FGA	4	0.01	0.71039	0.57731	0.85574	-0.04220	0.0757	0	18	1	2
FGA	4	0.001	0.76176	0.76176	0.76176	-0.04146	0.1368	0	4	0	2

CHR, chromosome; MAF, minor allele frequency; max MAF, this is the cutoff for the maximum MAF of the variants used in the gene set analysis; SKAT, sequence kernel association test; BETA, the beta coefficient is the degree of change in the outcome variable for every unit of change in the predictor variable; SE, standard error; MAC, minor allele count; Number rare, the number of variants with MAC above 10 in total; Number ultra rare, the number of variants with MAC below or equal to 10. These are combined in the analysis.
* The P-value for significance was set to 0.003125, according to Bonferroni adjustment for the number of tested genes (n=16).

Supplementary Table S6. Candidate gene set results for all cases of bleeding on factor Xa inhibitors (n=115) vs all controls (n=4891)

Region	CHR	max MAF	P value SKAT-O	P value Burden	P value SKAT	BETA Burden	SE Burden	MAC case	MAC control	Number rare	Number ultra rare
VWF	12	0.001	0.00171*	0.00171	0.00171	0.11150	0.0355	5	41	0	18
VWF	12	0.010	0.00762	0.11204	0.00428	0.02968	0.0187	9	206	6	18
VWF	12	0.100	0.09839	0.09009	0.06722	0.01898	0.0112	92	3816	15	18
VWF	12	0.400	0.09967	0.09240	0.06742	0.01855	0.0110	322	13470	19	18
VWF	12	0.200	0.09967	0.09240	0.06742	0.01855	0.0110	149	6199	17	18
FGG	4	0.001	0.04912	0.04912	0.04912	0.17339	0.0881	1	3	0	3
FGG	4	0.200	0.44291	0.84814	0.31482	-0.00704	0.0368	1	54	2	3
FGG	4	0.100	0.44291	0.84814	0.31482	-0.00704	0.0368	1	54	2	3
FGG	4	0.400	0.44291	0.84814	0.31482	-0.00704	0.0368	1	54	2	3
FGG	4	0.010	0.44291	0.84814	0.31482	-0.00704	0.0368	1	54	2	3
ABCB1	7	0.200	0.09629	0.05641	0.52373	-0.03628	0.0190	30	1914	6	11
ABCB1	7	0.400	0.09629	0.05641	0.52373	-0.03628	0.0190	30	1914	6	11
ABCB1	7	0.100	0.12495	0.07427	0.52934	-0.03419	0.0192	7	582	5	11
ABCB1	7	0.010	0.25091	0.15950	0.52430	-0.04449	0.0316	0	87	3	11
ABCB1	7	0.001	0.29192	0.29192	0.29192	-0.04621	0.0438	0	45	0	11
CES1	16	0.010	0.11997	0.12943	0.10134	0.05340	0.0352	4	80	1	2
CES1	16	0.400	0.40713	0.36848	0.35819	0.02633	0.0293	16	497	3	2
CES1	16	0.100	0.40713	0.36848	0.35819	0.02633	0.0293	16	497	3	2
CES1	16	0.200	0.40713	0.36848	0.35819	0.02633	0.0293	16	497	3	2
CES1	16	0.001	0.75053	0.75053	0.75053	-0.04181	0.1315	0	3	0	2
FGB	4	0.400	0.16405	0.43667	0.10293	0.01730	0.0222	52	1979	5	0
FGB	4	0.200	0.16405	0.43667	0.10293	0.01730	0.0222	52	1979	5	0
FGB	4	0.010	0.41102	0.54517	0.27174	0.01061	0.0175	4	92	4	0
FGB	4	0.100	0.41102	0.54517	0.27174	0.01061	0.0175	4	92	4	0
CES2	16	0.001	0.18914	0.18914	0.18914	0.10313	0.0785	1	6	0	3
CES2	16	0.200	0.47433	0.33324	0.39789	0.04035	0.0417	2	36	2	3
CES2	16	0.100	0.47433	0.33324	0.39789	0.04035	0.0417	2	36	2	3
CES2	16	0.400	0.47433	0.33324	0.39789	0.04035	0.0417	2	36	2	3

Region	CHR	max MAF	P value SKAT-O	P value Burden	P value SKAT	BETA Burden	SE Burden	MAC case	MAC control	Number rare	Number ultra rare
CES2	16	0.010	0.47433	0.33324	0.39789	0.04035	0.0417	2	36	2	3
CYP3A5	7	0.001	0.23588	0.23588	0.23588	0.08666	0.0731	1	13	0	4
CYP3A5	7	0.200	0.64381	0.46078	0.54407	0.01448	0.0196	8	269	4	4
CYP3A5	7	0.400	0.64381	0.46078	0.54407	0.01448	0.0196	8	269	4	4
CYP3A5	7	0.100	0.64381	0.46078	0.54407	0.01448	0.0196	8	269	4	4
CYP3A5	7	0.010	0.72779	0.73708	0.56503	0.01136	0.0338	2	66	2	4
F12	5	0.010	0.32762	0.22110	0.64342	-0.04347	0.0355	0	60	2	3
F12	5	0.400	0.32762	0.22110	0.64342	-0.04347	0.0355	0	60	2	3
F12	5	0.200	0.32762	0.22110	0.64342	-0.04347	0.0355	0	60	2	3
F12	5	0.100	0.32762	0.22110	0.64342	-0.04347	0.0355	0	60	2	3
F12	5	0.001	0.59824	0.59824	0.59824	-0.04229	0.0803	0	11	0	3
SULT1A1	16	0.100	0.35762	0.35762	0.35762	0.06026	0.0655	1	15	0	3
SULT1A1	16	0.200	0.35762	0.35762	0.35762	0.06026	0.0655	1	15	0	3
SULT1A1	16	0.010	0.35762	0.35762	0.35762	0.06026	0.0655	1	15	0	3
SULT1A1	16	0.001	0.35762	0.35762	0.35762	0.06026	0.0655	1	15	0	3
SULT1A1	16	0.400	0.35762	0.35762	0.35762	0.06026	0.0655	1	15	0	3
CYP3A4	7	0.100	0.37056	0.32781	0.25651	-0.02793	0.0285	1	125	2	3
CYP3A4	7	0.200	0.37056	0.32781	0.25651	-0.02793	0.0285	1	125	2	3
CYP3A4	7	0.400	0.37056	0.32781	0.25651	-0.02793	0.0285	1	125	2	3
CYP3A4	7	0.010	0.37056	0.32781	0.25651	-0.02793	0.0285	1	125	2	3
CYP3A4	7	0.001	0.61959	0.61959	0.61959	-0.04210	0.0848	0	10	0	3
SERPINF2	17	0.400	0.43297	0.32187	0.61629	-0.04340	0.0438	149	6526	4	7
SERPINF2	17	0.010	0.43447	0.32335	0.61520	-0.04335	0.0439	0	41	1	7
SERPINF2	17	0.100	0.43447	0.32335	0.61520	-0.04335	0.0439	0	41	1	7
SERPINF2	17	0.200	0.43553	0.32409	0.61613	-0.04321	0.0438	45	1916	2	7
SERPINF2	17	0.001	0.48589	0.48589	0.48589	-0.04346	0.0624	0	22	0	7
SERPINE1	7	0.100	0.43504	0.59166	0.31686	-0.01609	0.0300	32	1070	2	3
SERPINE1	7	0.400	0.43504	0.59166	0.31686	-0.01609	0.0300	32	1070	2	3
SERPINE1	7	0.200	0.43504	0.59166	0.31686	-0.01609	0.0300	32	1070	2	3

Region	CHR	max MAF	P value SKAT-O	P value Burden	P value SKAT	BETA Burden	SE Burden	MAC case	MAC control	Number rare	Number ultra rare
SERPINE1	7	0.001	0.62398	0.62398	0.62398	-0.04221	0.0861	0	10	0	3
SERPINE1	7	0.010	0.62398	0.62398	0.62398	-0.04221	0.0861	0	10	0	3
GGCX	2	0.100	0.46778	0.46778	0.46778	-0.04346	0.0598	0	20	0	7
GGCX	2	0.200	0.46778	0.46778	0.46778	-0.04346	0.0598	0	20	0	7
GGCX	2	0.001	0.46778	0.46778	0.46778	-0.04346	0.0598	0	20	0	7
GGCX	2	0.010	0.46778	0.46778	0.46778	-0.04346	0.0598	0	20	0	7
GGCX	2	0.400	0.46801	0.46791	0.46778	-0.04344	0.0598	77	3223	1	7
ABCG2	4	0.400	0.49440	0.59557	0.33395	0.01140	0.0215	43	1504	5	4
ABCG2	4	0.100	0.49440	0.59557	0.33395	0.01140	0.0215	43	1504	5	4
ABCG2	4	0.200	0.49440	0.59557	0.33395	0.01140	0.0215	43	1504	5	4
ABCG2	4	0.010	0.52876	0.45412	0.36755	0.02051	0.0274	4	120	3	4
ABCG2	4	0.001	0.63835	0.63835	0.63835	-0.04203	0.0894	0	9	0	4
THBD	20	0.400	0.51743	0.39211	0.62643	-0.04376	0.0511	45	2269	2	2
THBD	20	0.200	0.52881	0.40217	0.62647	-0.04286	0.0512	0	29	1	2
THBD	20	0.010	0.52881	0.40217	0.62647	-0.04286	0.0512	0	29	1	2
THBD	20	0.100	0.52881	0.40217	0.62647	-0.04286	0.0512	0	29	1	2
THBD	20	0.001	0.65660	0.65660	0.65660	-0.04195	0.0944	0	9	0	2
FGA	4	0.400	0.72808	0.59639	0.88626	-0.04256	0.0804	57	2627	2	2
FGA	4	0.010	0.73157	0.60019	0.88621	-0.04211	0.0803	0	18	1	2
FGA	4	0.100	0.73157	0.60019	0.88621	-0.04211	0.0803	0	18	1	2
FGA	4	0.200	0.73157	0.60019	0.88621	-0.04211	0.0803	0	18	1	2
FGA	4	0.001	0.77578	0.77578	0.77578	-0.04134	0.1451	0	4	0	2

CHR, chromosome; MAF, minor allele frequency; max MAF, this is the cutoff for the maximum MAF of the variants used in the gene set analysis; SKAT, sequence kernel association test; BETA, the beta coefficient is the degree of change in the outcome variable for every unit of change in the predictor variable; SE, standard error; MAC, minor allele count; Number rare, the number of variants with MAC above 10 in total; Number ultra rare, the number of variants with MAC below or equal to 10. These are combined in the analysis.

* The P-value for significance was set to 0.003125, according to Bonferroni adjustment for the number of tested genes (n=16).
